# Differential Proteomic Analysis of Noncardia Gastric Cancer from Individuals of Northern Brazil

**DOI:** 10.1371/journal.pone.0042255

**Published:** 2012-07-30

**Authors:** Mariana Ferreira Leal, Janete Chung, Danielle Queiroz Calcagno, Paulo Pimentel Assumpção, Samia Demachki, Ismael Dale Cotrim Guerreiro da Silva, Roger Chammas, Rommel Rodríguez Burbano, Marília de Arruda Cardoso Smith

**Affiliations:** 1 Genetics Division, Department of Morphology and Genetic, Federal University of São Paulo, São Paulo, Brazil; 2 Department of Microbiology, Immunology and Parasitology, Federal University of São Paulo, São Paulo, Brazil; 3 Surgery Service, Federal University of Pará, João de Barros Barreto University Hospital, Belém, Brazil; 4 Pathology Service, Federal University of Pará, João de Barros Barreto University Hospital, Belém, Brazil; 5 Department of Gynecology, Federal University of São Paulo, São Paulo, Brazil; 6 Laboratory of Experimental Oncology, School of Medicine, University of São Paulo, São Paulo, Brazil; 7 Human Cytogenetics Laboratory, Institute of Biological Sciences, Federal University of Pará, Belém, Brazil; Deutsches Krebsforschungszentrum, Germany

## Abstract

Gastric cancer is the second leading cause of cancer-related death worldwide. The identification of new cancer biomarkers is necessary to reduce the mortality rates through the development of new screening assays and early diagnosis, as well as new target therapies. In this study, we performed a proteomic analysis of noncardia gastric neoplasias of individuals from Northern Brazil. The proteins were analyzed by two-dimensional electrophoresis and mass spectrometry. For the identification of differentially expressed proteins, we used statistical tests with bootstrapping resampling to control the type I error in the multiple comparison analyses. We identified 111 proteins involved in gastric carcinogenesis. The computational analysis revealed several proteins involved in the energy production processes and reinforced the Warburg effect in gastric cancer. ENO1 and HSPB1 expression were further evaluated. ENO1 was selected due to its role in aerobic glycolysis that may contribute to the Warburg effect. Although we observed two up-regulated spots of ENO1 in the proteomic analysis, the mean expression of ENO1 was reduced in gastric tumors by western blot. However, mean ENO1 expression seems to increase in more invasive tumors. This lack of correlation between proteomic and western blot analyses may be due to the presence of other ENO1 spots that present a slightly reduced expression, but with a high impact in the mean protein expression. In neoplasias, HSPB1 is induced by cellular stress to protect cells against apoptosis. In the present study, HSPB1 presented an elevated protein and mRNA expression in a subset of gastric cancer samples. However, no association was observed between HSPB1 expression and clinicopathological characteristics. Here, we identified several possible biomarkers of gastric cancer in individuals from Northern Brazil. These biomarkers may be useful for the assessment of prognosis and stratification for therapy if validated in larger clinical study sets.

## Introduction

Gastric cancer (GC) is the fourth most common cancer and the second leading cause of cancer-related death worldwide [1]. The overall relative 5-year survival rate is currently less than 20% [2]. In Northern Brazil, GC is the second most frequent neoplasia among males and the third in females [3]. In Pará State, Northern Brazil, the 5-year survival rate is about 9–10% [4].

The two main tumor sites of GC are cardia (proximal) and noncardia (distal). The cardia GC affects five times more men than women [5]. In addition, the incidence rates of cardia GC are relatively high in the professional classes [6]. In contrast, the noncardia GC has a male-to-female ratio of approximately 2∶1 and the incidence rises progressively with age, with a peak incidence between 50 and 70 years [7]. The risk factors for noncardia GC include *Helicobacter pylori* infection, low socioeconomic status, smoking, intake of salty and smoked food, and low consumption of fruits and vegetables [8]. Over the last few decades, the incidence of noncardia GC has substantially declined in developed regions of the world. However, this subtype still constitutes the majority of GC cases worldwide and remains common in many geographic regions, including China, Japan, Eastern Europe and Central/South Americas [8]. The understanding of GC biology and the identification of cancer biomarkers are necessary to reduce the mortality rates through cancer screenings in high-risk populations, to increase early diagnosis, and to develop new target therapies.

GC, as other neoplasias, is thought to result from a combination of environmental factors and the accumulation of generalized and specific genetic and epigenetic alterations, which affect oncogenes, tumor suppressor genes, and control genomic instability. Several genes/proteins have been proposed as GC biomarkers. In the multistage gastric carcinogenesis, alterations of the oncogenes MYC, KRAS2, CTNNB1, ERBB2, FGFR2, CCNE1 and HGFR, as well as of the tumor suppressors TP53, APC, RB, DCC, RUNX3 and CDH1 have been so far reported (see reviews [9], [10]). Although the deregulation of these genes/proteins has been intensively studied in GC, a more complete profiling is necessary to understand the carcinogenesis process.

The last decade in life sciences was deeply influenced by the development of the “Omics” technologies (genomics, transcriptomics, proteomics, and metabolomics) which aim to depict a global view of biological systems and the understanding of the living cell [11]. Since proteins are ultimately responsible for the malignant phenotype, proteomic analyses may reflect the functional state of cancer cells, and therefore have distinct advantages over genomics and transcriptomics studies [12]. Moreover, proteins are currently the main target molecules of anticancer drugs [13].

Some proteomic-based studies were previously performed in human primary gastric tumors (see review [14]). However, most of these studies analyzed tumors of individuals from Asian population and, thus, may not reflect the distinct biological and clinical behaviors among GC processes. GC is marked by global variations in incidence, etiology, natural course, and management [15]. Although, about 90% of stomach tumors are adenocarcinomas [7], several factors lead to biologically and clinically GC subsets: antecedent tumorigenic conditions, such as *Helicobacter pylori* gastritis and other chronic gastric pathologies; location of the primary tumor (cardia and noncardia region); subtypes of adenocarcinoma (diffuse, intestinal, or mixed [16]); ethnicity of the afflicted population (differing levels of susceptibility and aggressiveness of the tumors); and a predictive biomarker (ERBB2) [15]. Thus, the term “gastric cancer” is used to describe several neoplasias that affect the stomach region.

In the present study, we compared the expression profile of noncardia GC and the matched non-neoplastic gastric tissue of individuals from Northern Brazil (all with *H. pylori* infection), and identified a protein signature that was differentially expressed between the two groups by a two-dimensional electrophoresis (2-DE) method. To screen proteins related to the progression of gastric carcinogenesis, we also compared non-neoplastic gastric tissues with GC samples of individuals with and without lymph node metastases. For the selection of differentially expressed proteins, we used a statistical parametrics test with bootstrapping resampling. We have undertaken a comprehensive computational analysis of tissue proteomic data to discover pathways and networks involved in gastric oncogenesis and progression. The possible associations among enolase 1 (ENO1) and heat shock 27 kDa protein 1 (HSPB1) gene and protein expression, and clinicopathological characteristics were also evaluated in individuals from Northern Brazil. These two proteins have been reported frequently as deregulated molecules in previous GC proteomic studies in other populations. However, they were never evaluated in a Brazilian population.

## Methods

### Clinical specimens

For protein profiling analysis, noncardia GC samples and corresponding non-neoplastic gastric tissue (distant location of primary tumor; control group) were collected from 15 patients. Additional paired samples were included for western blot and real time quantitative PCR (RT-qPCR) analyses. Dissected tissue specimens were snap-frozen in liquid nitrogen after surgical dissection and stored at −80°C until use. All the gastric samples were obtained surgically from João de Barros Barreto University Hospital (HUJBB) in Pará State, Northern Brazil. In Pará State, the human population is composed of interethnic crosses between three main origin groups: European (mainly represented by the Portuguese), Africans, and Amerindians [17]. All patients had negative histories of exposure to either chemotherapy or radiotherapy before surgery and there was no other co-occurrence of diagnosed cancers. Written informed consent with approval of the ethics committee of HUJBB was obtained from all patients prior to specimen collection.

All samples were classified according to Laurén [16] and tumors were staged using standard criteria by TNM staging [18]. The presence of *H. pylori*, a class I carcinogen, in gastric samples was detected by PCR assay for the urease gene [19] and for its virulence factor vacuolating cytotoxin (CagA) [20] using the DNA purified simultaneously with the proteins and the mRNA. In each PCR experiment, positive and negative controls were included.

To reduce sample heterogeneity, the 2-DE analysis included only noncardia GC and gastric samples presenting *H. pylori* infection CagA+, which seems to be frequent in our population (unpublished data). Table 1 shows the clinicopathological characteristics of samples used for protein profiling analysis.

**Table 1:** Clinicopathological characteristics of gastric cancer samples used for protein profiling analysis.

Patient	Gender	Age	Laurén classification	TNM	*H. pylori* – *UreA* a	*H. pylori* – *CagA* a
T1	Male	55	Intestinal	T1b-Sm	Positive	Positive
T2	Male	35	Intestinal	pT3N3M	Positive	Positive
T3	Female	73	Diffuse	pT1N0M	Positive	Positive
T4	Male	57	Diffuse	pT2N1M	Positive	Positive
T5	Female	55	Intestinal	pT4N1M	Positive	Positive
T6	Female	43	Intestinal	pT4N1M	Positive	Positive
T7	Male	68	Intestinal	pT3N1M	Positive	Positive
T8	Female	40	Intestinal	pT2N0M	Positive	Positive
T9	Female	50	Intestinal	pT1N0M	Positive	Positive
T10	Female	47	Intestinal	pT3N2M	Positive	Positive
T11	Female	23	Diffuse	pT3N1M	Positive	Positive
T12	Male	82	Intestinal	pT3N1M	Positive	Positive
T13	Female	79	Intestinal	pT1N0M	Positive	Positive
T14	Male	48	Intestinal	pT2N1M	Positive	Positive
T15	Male	72	Intestinal	pT3N0M	Positive	Positive

a *H. pylori* infection was also present in all corresponding non-neoplastic gastric samples.

### Protein, mRNA and DNA purification

Total protein, mRNA, and DNA were simultaneously isolated from gastric tissue samples using the AllPrep DNA/RNA/Protein Kit (Qiagen, Germany) according to the manufacturer's instructions. The protein pellet was dissolved in buffer containing 7 M urea, 2 M thiourea, 4% CHAPS, 50 mM DTT, 1% Protease Inhibitor Cocktail (Sigma), and 0.5% of each Phosphatase Inhibitor Cocktail 1 and 2 (Sigma-Aldrich, USA). Protein concentration was determined by the method of Bradford (Sigma-Aldrich, USA). RNA concentration and quality were determined using a NanoDrop spectrophotometer (Kisker, Germany) and 1% agarose gels. Samples were stored at −80°C until use.

### 2-DE proteomics profiling

Proteins were separated by 2-DE [21]. Protein samples (300 µg) were loaded on IPG strips (pH 3–10 L, 18 cm, GE Healthcare, USA). The strips were rehydrated for 12 h at 50 V. The isoelectric focusing was carried out using the following program: 200 V 2 h, 300 V 30 min, 500 V 30 min, 1000 V 1 h, 8000 V 1.5 h and 80,000 Vh. The focused strips were reduced with 50 mM DTT and alkylated with 100 mM iodoacetamide in buffer containing 6 M urea, 50 mM Tris-HCl (pH 8.8), 30% glycerol, 2% SDS, and trace bromophenol blue. For secondary electrophoresis, the treated strips were loaded on the top of 12.5% SDS-PAGE (200 mm) together with the PeppermintStick™ Phosphoprotein Molecular Weight Standards (Invitrogen, USA). In all experiments, GC and paired control samples were performed simultaneously. All 2-DE gels were performed in duplicate.

The proteins on the 2-DE gels were visualized using SYPRO® Ruby Gel Stain (Invitrogen, USA) following the manufacturer's recommendations. Images of gels stained with SYPRO® Ruby were scanned with a 582 nm excitation and a 610 nm 30 bandpass emission filter on a Typhoon Trio imager (GE Healthcare, USA). For spot excision and subsequent mass spectrometry analysis, the gels were visualized with Coomassie Brilliant Blue G250 staining.

### Image analysis and spot identification

Gel images were analyzed with the PDQuest Advanced software version 8.0 (Bio-Rad, USA), according to the manufacturer's protocol. Automatic spot detection in each gel was verified by visual inspection. Some spots were used as landmarks to align the images. The spot intensities were normalized to equalize the total densities of each gel image using the Local Regression Model. Normalized protein spot volumes in the proteomes were compared among experimental groups.

We created two sets of analyses to identify differentially expressed spots: (1) the comparison of protein expression between tumor and matched control using paired T-test; (2) comparison of protein expression among control samples, GC without lymph-node metastasis, and GC with lymph-node metastasis using One-Way Analysis of Variance for independent samples (one-way ANOVA). In addition, post-hoc comparisons were performed with Tukey's test for normally distributed variables and with Games-Howell for distributions where equal variances could not be assumed. The parametric tests analyses were performed with bootstrapping, a resampling method. The bootstrapping method [22] provides a critical adjusted p-value, controlling a type I error (false positive), reducing over-fit bias and validating the accuracy estimates. The bootstrapping technique can provide a more accurate and less conservative familywise error rate (FWER) than standard methods (e.g., Bonferroni's adjustment) for multicomparison analyses [23]. We performed all the analyses to identify differently expressed proteins based on 1000 bootstrap samples. In all analyses, the confidence interval (CI) was 95% and p values less than 0.05 were considered significant.

For protein identification by mass spectrometry, we filtered out significant spots presenting a fold change below 1.5-fold in the density between two experimental groups. These spots were observed in less than 30% of the samples in a group [24].

### Mass spectrometry analysis

The spots of interest were manually excised for subsequent mass spectrometry analysis [25]. In addition, blank gel pieces from a spot-free region, and reference spots (known marker proteins from the 2-DE) were excised, served as controls for trypsin digestion, and were run in parallel with the protein spots of interest. After destaining and washing, the gel particles were subjected to in-gel digestion with 20 ng/µl of Trypsin Gold (Mass Spectrometry Grade, Promega Corporation, USA) in 25 mM ammonium bicarbonate at 37°C overnight. Digested peptides were recovered, placed in a Speed-vac centrifuge until dry, and were reconstituted in 15 µl of 0.1% formic acid in water. The peptide mixture was analyzed using C18 ultra-performance liquid chromatography (UPLC, NanoAcquity, Waters, USA) coupled with ESI-quadrupole TOF mass spectrometer (ESI-QTOF Ultima, Waters/Micromass, USA). The gradient was 0–80% acetonitrile in 0.1% formic acid over 20 or 10 min. All MS/MS spectra were searched against the IPIhuman 379 database using MASCOT search engine (Matrix Science), accepting one missed tryptic cleavage and carbamidomethyl (C) as fixed modification and oxidation (M) as variable modifications. Peptide tolerance was ±0.1 Da and MS/MS tolerance was ±0.1 Da. Highest confidence identification had statistically significant search scores and protein identifications were based on a minimum of 2 peptide hits.

### Bioinformatics analysis

The identified proteins (from only the spots where one protein was identified) were classified into groups according to subcellular compartmentalization, biological process, and molecular function based on the information annotated in the Gene Ontology (GO) Consortium databases (http://www.geneontology.org/). This classification analysis was performed using the DAVID functional annotation tool (http://david.abcc.ncifcrf.gov/) [26]. The chromosomal location of identified proteins was accessed using BioMart data mining tool directly from Ensembl database (http://www.ensembl.org/biomart/martview).

The PANTHER system (www.pantherdb.org/), MetaCore software (GeneGo Inc.), and the Ingenuity Pathways Analysis 9.0 (IPA, Ingenuity Systems Inc.) were used for pathway, network, and functional analyses of differentially expressed proteins. All reported pathways and biological processes are listed according to their GO enrichment score provided by the software packages as −Log (p-values) and with a False Discovery Rate (FDR) of 0.05%.

The unsupervised clustering analysis was performed using Pearson's correlation of the normalized (z-score) values of all significant differentially expressed proteins (unique ID by spot) of 30 samples in the analysis set. Heat maps were generated using the Heatmap builder (http://ashleylab.stanford.edu/tools_scripts.html) to arrange protein expression profiles and samples on the basis of their similarity. Each colored cell on the two-dimensional map represents the protein expression value of the sample. Increasingly positive values are indicated by reds of increasing intensity, and increasingly negative values by greens of increasing intensity. Dendrograms on the top of the heat map show the clustering of samples and on the side of the clustering by protein expression.

### ENO1 and HSPB1 expression

The protein and mRNA used for the ENO1 and HSPB1 expression analyses were purified simultaneously with the protein sample used for 2-DE gels.

For western blot analysis, reduced protein (20 µg for ENO1 and 30 µg for HSBP1) of each sample was separated on 12.5% homogeneous SDS-PAGE gel and electro-blotted to a PVDF membrane (Hybond-P, GE Healthcare, USA). The PVDF membrane was blocked with phosphate-buffered saline containing 0.1% Tween 20, 5% low fat milk and incubated overnight at 4°C with corresponding primary antibodies to anti-ENO1 (sc-100812, 1∶3000, Santa Cruz Biotechnology, USA), anti-HSBP1 (sc-13132, 1∶1000, Santa Cruz Biotechnology, USA), and anti-ACTB (Ac-74, 1∶3000, Sigma-Aldrich, USA). After extensive washing, a peroxidase-conjugated secondary antibody was incubated for 1 h at room temperature. Immunoreactive bands were visualized using Western blotting Luminol reagent and the images were acquired using an ImageQuant 350 digital image system (GE Healthcare, Sweden). ACTB was used as a loading reference control.

For RT-qPCR analysis, the complementary DNA was synthesized using High-Capacity cDNA Archive kit (Applied Biosystems, Poland) according to the manufacturer's protocol. All real-time RT-qPCR assays were performed in triplicate for both target gene (*ENO1*: Hs00361415_m1; *HSPB1*: Hs03044127_g1, Applied Biosystems, USA) and internal controls (*ACTB*: Hs03023943_g1; *GAPDH*: Hs99999905_m1, Applied Biosystems, USA) using primers and TaqMan probes. Relative quantification (RQ) of the gene expression was calculated according to Pfaffl method [27]. A control sample was designated as a calibrator of each paired tumor.

### Statistical analysis of ENO1 and HSBP1 expression data

We first evaluated the normal distribution of all data using the Shapiro-Wilk normality test to determine subsequent use of appropriate tests for statistical comparison. *ENO1* mRNA levels and HSPB1 expression data were not normally distributed and were transformed (z-score) for analysis. Paired T-test was performed to compare the mean of ENO1 and HSPB1 protein expression between control and GC samples. The associations between clinicopathological parameters and the mean of ENO1 and HSPB1 expression were assessed using T-test for independent samples. Chi-square test (χ^2^) was also used to evaluate the relationship between expression data and clinicopathological factors. The correlation between the ENO1 and HSPB1 mRNA and protein expression (western blot and 2-DE analyses) was analyzed by Spearman test. In all analyses, the CI was 95% and p values less than 0.05 were considered significant.

## Results and Discussion

### Proteomic analysis of gastric tissue

**Figure 1:**
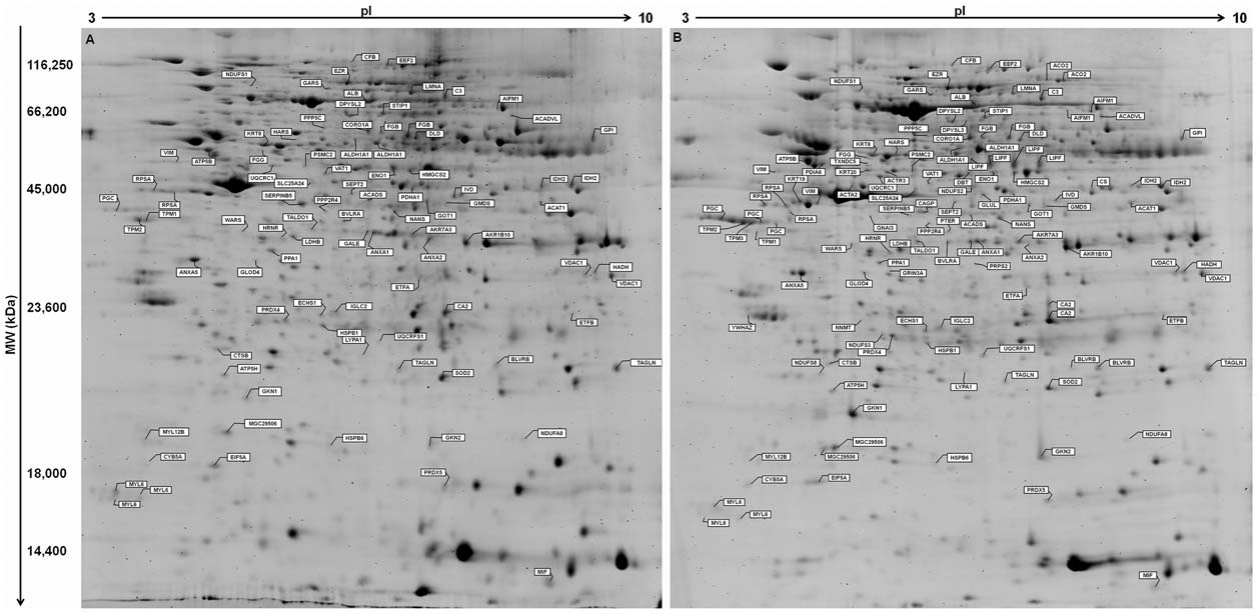
Representative 2-DE gel images of (A) gastric tumors and (B) non-neoplastic gastric samples. Proteins were resolved over the pI range 3–10, followed by 12.5% SDS-PAGE and stained with SYPRO® Ruby. The identified proteins that showed significantly altered expression in gastric carcinogenesis are labeled with the respective protein IDs.

We analyzed the proteome of 15 noncardia GC samples – 6 without lymph node metastasis and 9 with lymph node metastasis – and 15 matched control tissues (Table 1). Using 2-DE with pI range of 3.0–10 and molecular range between 10 kDa and 120 kDa, about 1000 spots were clearly detected by gel and subsequently analyzed for differential protein expression. Figure 1 shows representative gel images with the spots and their protein ID. Some proteins were reflected by multiple spots most likely due to posttranslational modification leading to shifts in the 2-DE gel. Details of the protein identifications, theoretical pI value, molecular weight, protein score, sequence coverage, number of matched peptides, as well as average relative change and p-values are shown in supplementary tables S1 and S2. These tables also show which proteins were previously observed in GC proteomic studies.

Although some proteomic-based studies were previously performed in human primary gastric tumors, to our knowledge, only three studies focus on noncardia GC [28], [29], [30]. Most GC proteomic studies identified differentially expressed proteins based only on a fold change between two conditions [24], [28], [30], [31], [32], [33], [34], [35]. Other previous GC proteomic studies performed statistical analyses to compare the protein expression between groups [29], [36], [37], [38], [39], but without controlling the type I (false positive) error. It is interesting to note that we compared the protein profiling of tumors and non-neoplastic samples using parametric tests with bootstrapping for differentially expressed protein identification. The resampling methods, commonly used in microarray studies [40], have been used to make p-value adjustments for multiple testing procedures which control the FWER and take into account the dependence structure between test statistics [41]. The objective is to create many sets of bootstrap samples by resampling with the replacements from the original data [22].

The comparison of tumor and control samples by paired T-test and the use of bootstrapping revealed 133 spots that were significantly altered with more than 1.5 fold change. Following a Mascot database search using the acquired MS data, 97% of the spots were identified. Among these spots, 18% were identified with more than one protein. The analyses of the spots with a unique identified proteins showed that 33 spots of 26 proteins were up-regulated and 72 of 56 proteins were down-regulated in GC samples compared to non-neoplastic tissue.

The comparison of tumor and control samples by one-way ANOVA revealed 143 differentially regulated spots and 98% of these were identified. We observed that 94 spots were common in paired T-test and ANOVA analyses. Concerning the spots with a unique identified protein, we observed that 54 proteins were down-regulated and 9 were up-regulated in tumor without lymph node metastasis relative to control samples. We also observed that 68 proteins were down-regulated and 15 were up-regulated in tumors with lymph node metastasis compared to control samples. We detected 38 proteins differentially expressed between non-neoplastic samples and tumors independent of the lymph node status.

Fourteen proteins presented significant alterations between tumors with and without lymph node metastasis (Figure S1). The expression of NNMT continually increased while ATP5H and UQCRFS1 showed a continuous reduction from non-neoplasia to tumorigenesis with lymph node metastasis. NNMT was previously reported with elevated expression in GC compared to non-neoplastic samples [24], [42]. The reduced expression of ATP5H and UQCRFS1 may be due to a lower dependence of oxidative phosphorylation for energy production, due to the Warburg effect in advanced state (see bioinformatics results below).

### Functional classification of differentially expressed proteins in GC

**Figure 2:**
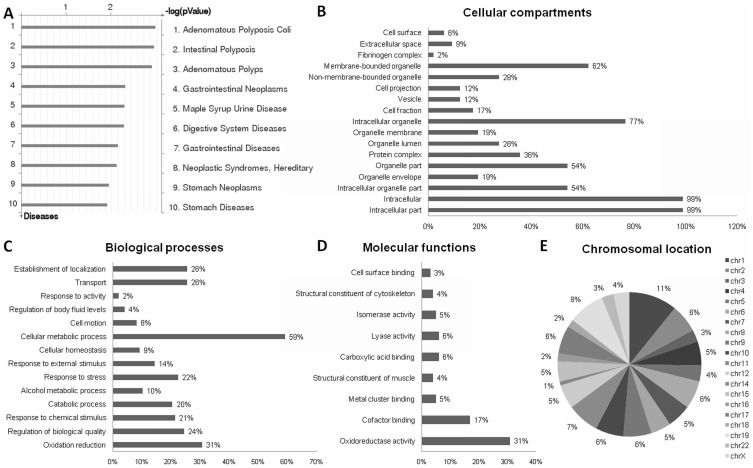
The identified proteins were grouped into different classes. A) Enrichment of GeneGo disease using the differentially regulated proteins between neoplastic and matched non-neoplastic samples; B) Cellular compartments, C) Biological processes, D) Molecular functions and E) Chromosomal location of all identified proteins.

The GeneGo biomarker analysis revealed that most of the identified proteins predicted markers for gastrointestinal diseases, including GC (Figure 2A, representative of tumor versus nontumor comparison). This supports the current data and indicates that the findings should be further investigated to identify or validate clinically relevant targets for the diagnosis and/or the prognosis of GC.

The identified proteins were grouped into different classes based on functional information available. Most of the identified proteins were intracellular organelle proteins and were part of the membrane-bound organelles, especially the mitochondria (Figure 2B). These proteins are involved mainly in cellular metabolic processes and oxidation reduction (Figure 2C). The comparison of tumors with lymph node metastases and control samples revealed the transport and the establishment of location as enriched biological processes. This was not observed in the comparison of tumors without lymph node metastases and control samples. The oxidoreductase activity followed by coenzyme binding activity were the main molecular functions of the proteins involved in gastric carcinogenesis (Figure 2D).

Concerning the chromosomal location, most of the proteins were encoded by genes located at chromosome 1 (11%), chromosome 19 (8%), and chromosome 11 (7%) (Figure 2E). Complex karyotypes of gastric tumors preferentially involve these chromosomes, as well as chromosomes 3, 6, 7, 8, 13 and 17 (see review [9]). Gain and loss of chromosome regions of 1, 19 and 11 were previously reported by our research group in GC samples [43], [44]. Thus, these chromosomes may contain several loci with dosage-sensitive genes.

### Networks analysis of differentially expressed proteins in GC

We have undertaken a comprehensive computational analysis of tissue proteomic data to discover pathways and networks involved in gastric oncogenesis and progression. Table 2 shows the principal canonical pathways using the Ingenuity Pathway Analysis (IPA) database. The principal canonical pathways in the gastric carcinogenesis process were involved in the energy metabolism pathways including mitochondrial dysfunction, pyruvate metabolism, oxidative phosphorylation, citrate circle, and glycolysis/gluconeogenesis. The present study demonstrated a large number of differential expressed metabolic proteins that have not been reported before in noncardia gastric carcinogenesis (Table S1 and S2).

**Table 2:** Top canonical pathways by Ingenuity Pathways Analysis.

Top canonical pathways		p-value*	Proteins
Tumor *vs* non-neoplastic samples	Mitochondrial Dysfunction	5.01E-11	PDHA1, NDUFS1, ATP5B, NDUFS8, PRDX5, NDUFS2, UQCRFS1, UQCRC1, NDUFS3, NDUFA8, AIFM1
	Pyruvate Metabolism	1.54E-10	PDHA1, AKR7A3, ALDH1A1, LIPF, AKR1B10, ACAT1, DLD, PDHB, LDHB
	Oxidative Phosphorylation	2.88E-09	NDUFS1, ATP5B, NDUFS8, ATP5H, NDUFS2, UQCRFS1, UQCRC1, NDUFS3, PPA1, NDUFA8
	Valine, Leucine and Isoleucine Degradation	4.26E-08	ALDH1A1, BCAT2, ACADVL, ACAT1, DBT, IVD, HADH
	Butanoate Metabolism	5.52E-07	PDHA1, ALDH1A1, ACAT1, DBT, PDHB, HADH
	Glycolysis/Gluconeogenesis	1.05E-05	PDHA1, ALDH1A1, ENO1, DLD, PDHB, LDHB
	Citrate Cycle	1.32E-05	LIPF, ACO2, DLD, IDH2
Tumor with lymph node metastasis *vs* non-neoplastic samples	Mitochondrial Dysfunction	1.67E-13	PDHA1, NDUFS1, SOD2, ATP5B, NDUFS8, PRDX5, NDUFS2, UQCRFS1, UQCRC1, NDUFS3, NDUFA8, AIFM1
	Oxidative Phosphorylation	3.88E-10	NDUFS1, ATP5B, NDUFS8, ATP5H, NDUFS2, UQCRFS1, UQCRC1, NDUFS3, PPA1, NDUFA8
	Butanoate Metabolism	4.9E-09	PDHA1, ALDH1A1, ECHS1, ACAT1, DBT, HADH, ACADS
	Valine, Leucine and Isoleucine Degradation	1.05E-08	ALDH1A1, ECHS1, BCAT2, ACAT1, DBT, HADH, ACADS
	Pyruvate Metabolism	7.21E-07	PDHA1, AKR7A3, ALDH1A1, LIPF, AKR1B10, ACAT1
	β-alanine Metabolism	2.19E-06	DPYSL2, ALDH1A1, ECHS1, DPYSL3, ACADS
	Lysine Degradation	3.16E-06	ALDH1A1, ECHS1, ACAT1, DBT, HADH
Tumor without lymph node metastasis *vs* non-neoplastic samples	Pyruvate Metabolism	1.13E-10	PDHA1, AKR7A3, ALDH1A1, LIPF, AKR1B10, ACAT1, DLD, PDHB
	Mitochondrial Dysfunction	2.94E-10	PDHA1, NDUFS1, NDUFS8, PRDX5, NDUFS2, UQCRFS1, UQCRC1, NDUFA8, AIFM1
	Butanoate Metabolism	8.22E-10	PDHA1, ALDH1A1, ACAT1, DBT, HMGCS2, PDHB, HADH
	Valine, Leucine and Isoleucine Degradation	1.77E-09	ALDH1A1, BCAT2, ACADVL, ACAT1, DBT, HMGCS2, HADH
	Citrate Cycle	3.29E-08	CS, LIPF, ACO2, DLD, IDH2
	Oxidative Phosphorylation	3.98E-07	NDUFS1, NDUFS8, ATP5H, NDUFS2, UQCRFS1, UQCRC1, NDUFA8
	Glycolysis/Gluconeogenesis	7.41E-07	PDHA1, GPI, ALDH1A1, ENO1, DLD, PDHB
Tumor with lymph node metastasis *vs* Tumor with lymph node metastasis	Pentose Phosphate Pathway	5.57E-04	TALDO1, GPI
	Purine Metabolism	2.71E-03	ATP5H, PPP2R4 PSMC2
	Oxidative Phosphorylation	8.34E-03	ATP5H, UQCRFS1

* The p-value was calculated using the right-tailed Fisher's Exact Test. Threshold: p<0.05.

Our proteomic analysis revealed that several enzymes of the citrate cycle (Krebs cycle) and of oxidative phosphorylation were down-regulated in GC cells. These data show possible alterations in mitochondrion function and a shift in energy production in the present GC cells, suggesting the Warburg effect [45]. Proliferating tumor cells reprogram their metabolic pathways to generate energy and, thus, support the rapid cell division under stressful metabolic conditions that are characteristic of the abnormal tumor microenvironment [46]. Even under normal oxygen concentrations, tumor cells shift from ATP generation through oxidative phosphorylation to ATP generation through glycolysis, converting most incoming glucose to lactate [45]. It has been proposed that highly active glycolysis provides a biosynthetic advantage for tumor cells. Glycolysis provides enough metabolic intermediates by avoiding the oxidation of glucose, which is essential for the synthesis of macromolecules, such as lipids, proteins, and nucleic acids, during cell division [47], [48], [49].

The lactate dehydrogenase (LDH) and pyruvate dehydrogenase (PDH) complexes control the metabolism of pyruvic acids, transforming to either lactic acids or acetyl-CoA then entering the citrate cycle. The down-regulation of subunits of the LDH and PDH complexes suggests a reduction in pyruvate flux into the citrate cycle and a decrease in the rate of oxidative phosphorylation and oxygen consumption, reinforcing the glycolytic phenotype. Other down-regulated proteins, such as LIPF and GOT1, highlight the activation of other metabolic pathways with the impairment of the citrate cycle and oxidative phosphorylation. Several metabolic alterations that we observed in noncardia GC were also described by Cai *et al.* [36] in a cardia GC proteomic study, suggesting that these metabolic alterations are not specific to a GC subtype based on tumor location.

By the PANTHER system, the most significantly enriched pathway is the p53 pathway observed in the comparison between tumor and control samples. Additionally to its function in the DNA damage response and apoptosis, p53 is also a regulator of cell metabolism [50]. p53 promotes oxidative phosphorylation [51] and also inhibits the glycolytic pathway by up-regulating the expression of TP53-induced glycolysis and the apoptosis regulator (TIGAR) [52]. Therefore, the loss of p53 contributes to the acquisition of glycolytic phenotype. The loss of *TP53* locus is a common finding in GC of individuals from Northern Brazil [53]. Moreover, 20% of the GC analyzed by 2-DE presented p53 immunoreactivity (data not shown). The p53 immunoreactivity usually depends on accumulation of mutated proteins in the cell, which leads to a longer half-life [54].

The top networks of molecular interactions and functions were also identified using the IPA software (Figure S2; Table 3; Subnetwork from MetaCoreTM analysis were not shown). We showed several differently regulated proteins involved in cellular assembly and organization, and in inflammatory processes. The cellular assembly and organization was the principally enriched network observed in the comparison between controls and tumors with lymph node metastasis. Therefore, our data reveal that the molecules of the described subnetwork are important to the process of metastasis in noncardia gastric carcinogenesis.

**Table 3:** Top networks involved by Ingenuity Pathways Analysis.

Top network of molecular interactions and functions		Score	Focus proteins
Tumor *vs* non-neoplastic samples	Cancer, Reproductive System Disease, Genetic Disorder	43	23
	Cellular Assembly and Organization, Energy Production, Nucleic Acid Metabolism	39	19
	Lipid Metabolism, Small Molecule Biochemistry, Cardiovascular Disease	29	15
	Cell Death, Cancer, Cellular Development	17	10
	Free Radical Scavenging, Inflammatory Disease, Respiratory Disease	13	8
Tumor with lymph node metastasis *vs* non-neoplastic samples	Cellular Assembly and Organization, Genetic Disorder, Neurological Disease	50	22
	Organismal Injury and Abnormalities, Inflammatory Disease, Respiratory Disease	30	15
	Cell Death, Antigen Presentation, Cell-To-Cell Signaling and Interaction	27	14
	Cell Death, Gastrointestinal Disease, Hepatic System Disease	25	13
	Cardiac Arteriopathy, Cardiovascular Disease, Genetic Disorder	2	1
Tumor without lymph node metastasis *vs* non-neoplastic samples	Genetic Disorder, Respiratory Disease, Inflammatory Disease	30	14
	Decreased Levels of Albumin, Cellular Assembly and Organization, Dermatological Diseases and Conditions	27	13
	Energy Production, Nucleic Acid Metabolism, Small Molecule Biochemistry	24	12
	Lipid Metabolism, Nucleic Acid Metabolism, Small Molecule Biochemistry	24	12
	Cardiac Arteriopathy, Cardiovascular Disease, Genetic Disorder	2	1
Tumor with lymph node metastasis *vs* Tumor with lymph node metastasis	Cellular Compromise, Cell Death, Infection Mechanism	42	14

Previous studies have demonstrated that GC is strongly linked to chronic inflammation, and that infection with *H. pylori* may trigger the chronic inflammation that can lead to malignancy (see review [55]). However, the exact mechanism of this process is still not known. The identified proteins add new pieces to this process in gastric carcinogenesis.

### Hierarchical clustering of gastric samples

The unsupervised hierarchical clustering of the differentially expressed proteins revealed that the tumors and control samples do not form two distinct separate clusters (Figure S3 and S4). Although, hierarchical clustering revealed one group composed by only controls, the other group presented all tumor samples and two misclassified control samples. Molecular alterations may already exist in the misclassified non-neoplastic samples resulting from a complex interaction between *H. pylori*, including environmental and host-genetic factors.

The group composed by tumor samples seems to present two subgroups according to the protein expression profiling. However, no association with clinicopathological characteristic was observed.

### ENO1 expression in gastric tissue

We selected ENO1 and HSPB1 genes/proteins for further investigations. These proteins were present in the main network by IPA analysis of differentially expressed proteins between tumor and control samples (Figure S2-A) and were never evaluated in GC of Brazilian individuals. Moreover, according to the IPA database, ENO1 and HSPB1 could interact with MYC, p53 and 14-3-3 epsilon proteins (data not shown), that are frequently deregulated in GC samples of individuals from Northern Brazil [53], [56], [57], [58], [59], [60], [61].

ENO1 was also selected due to its role in the synthesis of pyruvate [62]. The overexpression of ENO1 is associated with tumor development through the aerobic glycolysis and it has been described in several tumor types (see review [63]). Additionally, ENO1 (48 kDa) is encoded by a gene which also encodes a *MYC* promoter-binding protein (MBP1, 37 kDa), using an alternative start codon. MBP1 associates with ENO1 to inhibit the transcription of the *MYC* oncogenes [63]. Interestingly, the MYC oncogene seems to collaborate with HIF1, a transcription factor responsible for gene expression during the cellular response to low oxygen conditions, in the activation of several glucose transporters and glycolytic enzymes, contributing to the Warburg effect [46]. In the present study, MYC immunoreactivity was observed in 67% of samples (data not shown).

**Figure 3:**
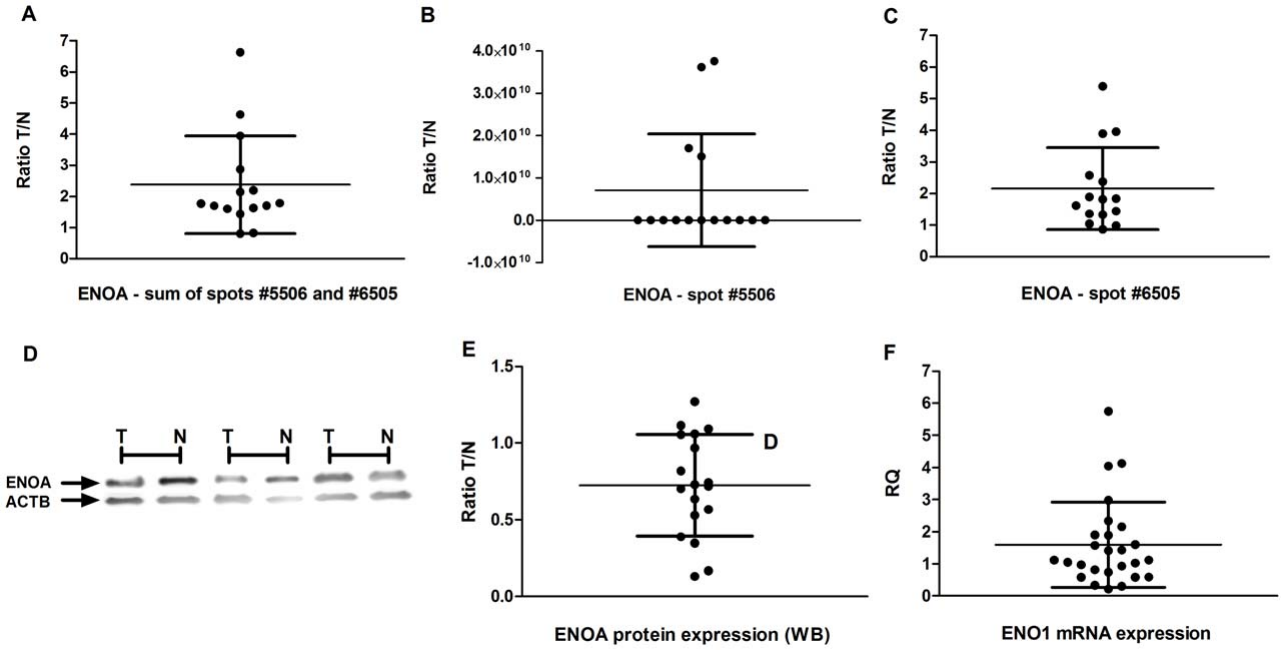
ENO1 expression in gastric samples. A) the ratio of the sum of spot #5506 and #6505 ENO1 expression between tumor and matched controls; B) the ratio of Spot #5506 ENO1 expression between tumor and matched controls; C) the ratio of spot #6505 ENO1 expression between tumor and matched controls; D) Western blot using anti-ENO1 and anti-ACTB antibodies; E) the ratio of ENO1 protein expression between tumor and matched controls by western blot analysis; F) Relative *ENO1* mRNA quantification – gastric tumor samples normalized by matched controls. T: tumor gastric sample; N: non-neoplastic gastric samples. *To calculate the ratio, 0 values (lack of a spot on the 2-DE gel) were replaced with 0.0001.

By 2-DE analysis, we observed two spots for the ENO1 (#5506 and #6505) protein that presented a higher expression in tumors compared to the controls (Figure 3A, 3B and 3C). Three previous proteomic studies also reported the up-regulation of ENO1 in GC samples [28], [29], [36] and one study described that ENO1 was down-regulated [39]. Moreover, it was previously demonstrated that ENO1 overexpression blocks gastrokine 1 (GKN1) induced growth inhibition and cell cycle arrest in gastric cancer cells [64].

Here, we showed the presence of two differentially expressed spots of ENO1 mainly in noncardia tumor samples, which suggests PTM. Spot #6505 presented a higher expression in both tumors with and without lymph node metastasis compared to the controls. However, spot #5506 only differed between tumors with lymph node metastases and controls. Cai *et al.* [36] also reported the up-regulation of two spots of ENO1 in cardia GC. PTM of ENO1 has been reported in several cancers and normal cell lines (see review [63]). Capello *et al.* [63] suggested that PTMs are important mechanisms in the regulation of ENO1 function, localization, and immunogenicity.

Inversely to the 2-DE observation, the protein level of ENO1 showed a 1.5 fold reduction in 35.3% of GC samples compared to their paired controls (Figure 3E and 3F) by western blot. Only one sample presented a 1.5 fold increase. However, we only selected the spots differentially expressed with a 1.5 fold change between groups for the mass spectrometry analysis. These selection criteria may lead to the lack of correlation between western blot and proteomic analyses. Thus, other spots of ENO1 may present a slight reduced expression, but with a high impact in the mean of this protein expression. Our results show that different spots may be regulated differently inside a heterogeneous gastric sample. Our findings also highlight that the metabolic phenotype is not universal in tumor cells [65], especially considering that different cell clones are present inside a single cancer sample. Even in glycolytic tumors, oxidative phosphorylation is not completely shut down. Owing to the dynamic nature of the tumor microenvironment, it is suggested that the metabolic phenotype of tumor cells changes to adapt to the prevailing local conditions [46]. The regulation of this metabolic flexibility is poorly understood. However, the feedback control between *MYC* and ENO1, as well as MBP1, may have a key role in this process since the *MYC* oncogene may stimulate both glycolysis and oxidative phosphorylation.

In the present study, the mRNA level of *ENO1* showed a 1.5 fold reduction in 35.3% and increased in 58.8% of GC samples compared to their paired control (Figure 3G). No correlation was observed between the ENO1 mRNA and protein level detected by western Blot (ρ = 0.219; p = 0.397). The mRNA level was correlated only to the expression of ENO1 spot #6505 by 2-DE analysis (ρ = 0.378, p-0.043). However, the *ENO1* gene encodes ENO1 and MBP1 proteins. The *MBP1* cDNA shares 97% similarity with the cDNA encoding the isoform of the glycolytic enzyme enolase [66]. Thus, the analysis of *ENO1* mRNA expression reflects only in part the ENO1 expression. On the other hand, Cai *et al.* [36] reported an increase of ENO1 mRNA and protein expression in cardia GC samples by RT-qPCR (primers for cDNA of ENO1 and MBP1) and western blot, but no statistical analysis was performed, making it difficult to formulate a direct comparison with our results.

Table 4 summarizes the associations between clinicopathological characteristics and ENO1 expression. The 1.5 fold reduction of ENO1 protein expression was more frequently observed in tumors in the T1/T2 stage than the T3/T4 stage (71.4% *vs* 28.6%; χ^2^ = 5.103, df = 1, p = 0.039; OR = 11.25). Few studies are aimed to a better understanding of the role of ENO1 in gastric carcinogenesis. Bai *et al.* [28], who described an up-regulated spot of ENO1, did not find a significant difference between tumor and non-neoplastic samples by western blot analysis. However, these authors described that ENO1 immnuoreactivity seems to be significantly more intense in GC cells than non-neoplastic cells and its positive expression tends to be associated with poor prognosis. This in part corroborates our results that demonstrate that the level of ENO1 protein seems to be reduced more frequently in less invasive cancer samples.

**Table 4:** Clinicopathological characteristics, ENO1 and HSPB1 expression in gastric cancer samples.

	ENO1 protein			*ENO1* mRNA			HSPB1 protein			*HSPB1* mRNA		
Variable	N	Ratio T/N (Mean±SD)	p-value	N	RQ (Mean±SD)	p-value	N	Ratio T/N (Mean±SD)	p-value	N	RQ (Mean±SD)	p-value
**Gender**												
Male	8	0.63±0.41	0.302	14	1.84±1.52	0.327	9	18.17±31.02	0.504	18	1.64±1.28	0.803
Female	10	0.80±0.63		12	1.31±1.05		10	10.32±18.06		13	1.88±3.667	
**Onset (years)**												
<45	5	0.68±0.24	0.762	7	1.21±0.75	0.386	5	20.10±42.21	0.691	8	1.09±0.55	0.403
≥45	13	0.74±0.37		19	1.74±1.48		14	11.87±16.53		23	1.97±2.89	
**Tumor location**												
Cardia	2	0.52±0.25	0.383	3	1.02±0.76	0.438	3	34.98±52.68	0.500	4	0.92±0.65	0.495
Non-cardia	16	0.75±0.34		23	1.67±1.38		16	10.11±15.95		27	1.86±2.68	
**Histological subtype**												
Diffuse-type	4	0.48±0.28	0.103	10	1.44±1.01	0.651	4	1.89±1.26	0.279	13	1.43±1.47	0.572
Intestinal-type	14	0.79±0.32		16	1.69±1.52		15	17.27±26.96		18	1.96±3.09	
**Stage**												
Early	4	0.57±0.40	0.308	4	1.09±0.56	0.421	4	6.87±7.69	0.529	4	1.24±0.80	0.678
Advanced	14	0.77±0.31		22	1.69±1.41		15	15.94±27.37		27	1.82±2.69	
**Tumor invasion**												
T1/T2	7	0.56±0.37	0.910	8	1.15±0.62	0.259	7	4.51±6.21	0.114	9	1.33±0.70	0.567
T3/T4	11	0.83±0.27		18	1.80±1.52		12	19.59±29.68		22	1.91±2.96	
**Lymph node metastasis**												
Absent	6	0.75±0.43	0.798	7	0.92±0.49	0.119	6	5.24±6.50	0.304	8	1.07±0.69	0.391
Present	12	0.71±0.29		19	1.84±1.46		13	18.09±28.92		23	1.97±2.88	
**Distant metastasis**												
Unknown/absent	15	0.71±0.35	0.746	20	1.39±0.93	0.361	15	14.15±27.30	0.955	23	1.39±1.17	0.432
Present	3	0.78±0.25		6	2.30±2.18		4	13.60±12.83		8	2.76±4.62	

RQ: relative quantification; T: tumor gastric samples; N: non-neoplastic gastric samples.

### HSBP1 expression in gastric tissue

HSPB1 was selected for further investigation also due to its protective function against infection and cellular stress. HSPB1 is one member of the family of heat shock proteins (HSP) that is characterized as molecular chaperones. In addition to its chaperone function, HSPB1 also seems to be an important regulator of structural integrity and membrane stability, actin polymerization and intermediate filament cytoskeleton formation, cell migration, epithelial cell-cell adhesion, cell cycle progression, proinflammatory gene expression, muscle contraction, signal transduction pathways, mRNA stabilization, presentation of oxidized proteins to the proteasome, differentiation, and apoptosis [67].

HSPB1 is highly induced by different stresses such as heat, oxidative stress, or anticancer drugs. In non stressed cells, HSPB1 is not expressed or at very low levels. Once induced, HSPB1 acts at multiple points in the apoptotic pathways to ensure that stress-induced damage does not inappropriately trigger cell death [68]. Many cancer cells have markedly increased HSPB1 levels, and this protein expression contributes to the malignant properties of these cells, including increased tumorigenicity and treatment resistance, and apoptosis inhibition [67]. Overexpression of HSPB1 has been described in several tumors and it has been reported as an indicator of poor prognosis (see review [69]). Elevated HSPB1 expression in neoplastic cells plays a key role in protection from spontaneous apoptosis in response to anticancer therapy and leading to tumor progression and resistance to treatment [69].

**Figure 4:**
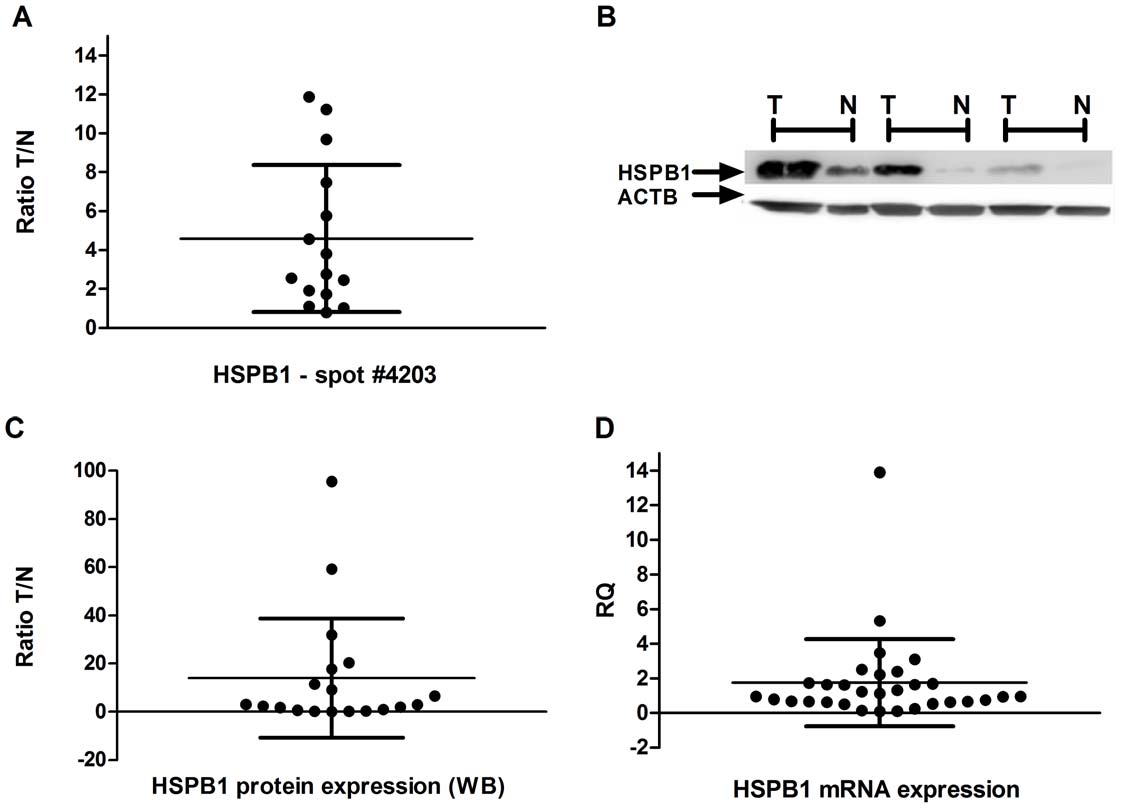
HSPB1 expression in gastric samples. A) the ratio of spot #4203 HSPB1 expression between tumor and matched controls; B) western blot using anti-HSPB1 and anti-ACTB antibodies; C) the ratio of HSPB1 protein expression between tumor and matched controls by western blot analysis; D) Relative HSPB1 mRNA quantification – gastric tumor samples normalized by matched controls. T: tumor gastric sample; N: non-neoplastic gastric samples.

In the present study, we observed one spot of the HSPB1 protein that presented a higher expression in GC compared to controls by 2-DE analysis (Figure 4A), corroborating previously proteomic studies with GC patients from Asiatic countries [24], [28], [33], [34], [35], [36], [39].

By Western blot analysis, the protein level of HSPB1 was 1.5 fold higher in 68.4% of GC samples compared to their paired controls and 1.5 fold lower in 21.1% of the samples (Figure 4B and 4C). The *HSPB1* mRNA level was 1.5 fold higher in 38.7% and reduced in 32.3% of tumors compared to controls (Figure 4D). Previous studies also described a high expression of HSPB1 in about 50% of GC by immunohistochemistry in other populations [70], [71], [72], [73].

The increase in the *HSPB1* mRNA level was correlated with the protein expression observed by 2-DE analysis (ρ = 0.601, p = 0.018). Additionally, we observed a correlation between the increase of 1.5 fold in mRNA and protein levels by western blot (ρ = 0.54; p = 0.025). The lack of strong correlation between HSPB1 protein and mRNA expression patterns indicates the post-translational regulation mechanism involved in this protein expression and highlights the complexity of the relationship between protein and mRNA expression.

No correlation was observed between the HSPB1 protein level detected by western blot and 2-DE analysis (ρ = 0.075; p = 0.792). However, it is possible to verify that the fold changes of HSPB1 by 2-DE analysis (range of 0.79 to 11.86) is lower than that observed by western blot analysis (range of 0.15 to 95.59), suggesting that the other HSPB1 isoforms (with the same molecular weight) are also recognized by the anti-HSPB1 monoclonal antibody. These isoforms may be differentially regulated in GC of individuals from Northern Brazil, which may lead to the lack of correlation between these two methodologies. This hypothesis is supported by Cai *et al.* in a study [36] in which two differentially regulated spots of HSPB1 were detected in cardia GC. The authors observed that the mean of HSPB1 expression was elevated in tumors compared to non-neoplastic tissue.

Here, no association was observed among HSPB1 expression and clinicopathological characteristics in the present study (Table 4). However, HSPB1 was previously associated with gastric tumor size, distant metastasis, lymph node state and pStage in other populations [70], [71], [72], [73]. Despite the fact that HSPB1 expression was not associated with any clinicopathological characteristic in our population, we hypothesized that HSPB1, especially the isoform detected by 2-DE analysis, may have a role in the carcinogenesis process in a subset of tumors due to the higher expression observed in several GC samples.

In addition, since HSPB1 contributes to chemotherapy resistance and apoptosis inhibition in gastric cancer cells [74], the high levels of HSPB1 observed in our GC sample might be associated with anticancer drug resistance or survival, as well as poor patient prognosis. The lack of additional information about the survival or response to any adjuvant treatment from the studied patients is one limitation of this study.

### Conclusion

Our differential proteomic analysis revealed several potential proteins that are deregulated in noncardia GC of individuals from Northern Brazil. For the identification of differentially expressed proteins we controlled the type I error that is a main issue in multiple comparison analyses using bootstrapping resampling. The cancer-associated proteins could be useful for GC diagnosis or prognosis. Several identified proteins reinforce the Warburg effect, suggesting active glycolysis in neoplastic cells. Therapeutic approaches targeting glycolytic process may be an interesting future for GC treatment. On the other hand, the analysis of ENO1 expression highlights that the metabolic phenotype may be dynamic in GC samples. Although further investigations are necessary, HSPB1 may have a role in a subset of GC samples. Our results also underline the complex control of mRNA and protein expression. The present study will enhance efforts to generate and expand knowledge about gastric carcinogenesis and in doing so, aid in the discovery of more reliable diagnosis for malignancies of the stomach in the Brazilian population.

## Supporting Information

Figure S1 **14 significantly different proteins between tumors with and without lymph node metastasis.** The normalized means (z-scores) of the expression in non-neoplastic, neoplastic without [T(N−)] and with lymph node metastasis [T(N+)] is presented.
(TIF)

Figure S2 **Protein-protein physical/functional interaction subnetworks in gastric carcinogenesis by generated Ingenuity Pathway Analysis tool.** A) Cancer subnetwork revealed in the analysis of differentially expressed proteins between neoplastic and matched controls; B) Cellular assembly and organization, energy production, nucleic acid metabolism subnetwork revealed in the analysis of differentially expressed proteins between neoplastic and matched controls; C) Cellular assembly and organization subnetwork revealed in the analysis of differentially expressed proteins between controls and tumors with lymph node metastasis; D) Inflammatory subnetwork revealed in the analysis of differentially expressed proteins between controls and tumors without lymph node metastasis. Red: up-regulated proteins; Green: down-regulated proteins.
(TIF)

Figure S3 **Differentially expressed by paired T-test analysis. Heat map represents the expression protein level.** The samples are shown vertically and the proteins horizontally. Higher expressions are colored red, the lower ones in green. The dendrograms represent the distances between the clusters.
(TIF)

Figure S4 **Differentially expressed by one-way ANOVA analysis. Heat map represents the expression protein level.** The samples are shown vertically and the proteins horizontally. Higher expressions are colored red, the lower ones in green. The dendrograms represent the distances between the clusters.
(TIF)

Table S1 **Differentially expressed proteins between neoplastic and non-neoplastic gastric samples by paired T-test analysis.**
(DOCX)

Table S2 **Differentially expressed proteins among non-neoplastic gastric samples, neoplastic without lymph node metastasis and neoplastic with lymph node metastasis by ANOVA one-way analysis.**
(DOCX)
