# Supplementary figures and images for: Differential Proteomic Analysis of Noncardia Gastric Cancer from Individuals of Northern Brazil
Source: PLoS One. 2012 Jul 30;7(7):e42255. doi: 10.1371/journal.pone.0042255 (PMC3408468; doi:10.1371/journal.pone.0042255)

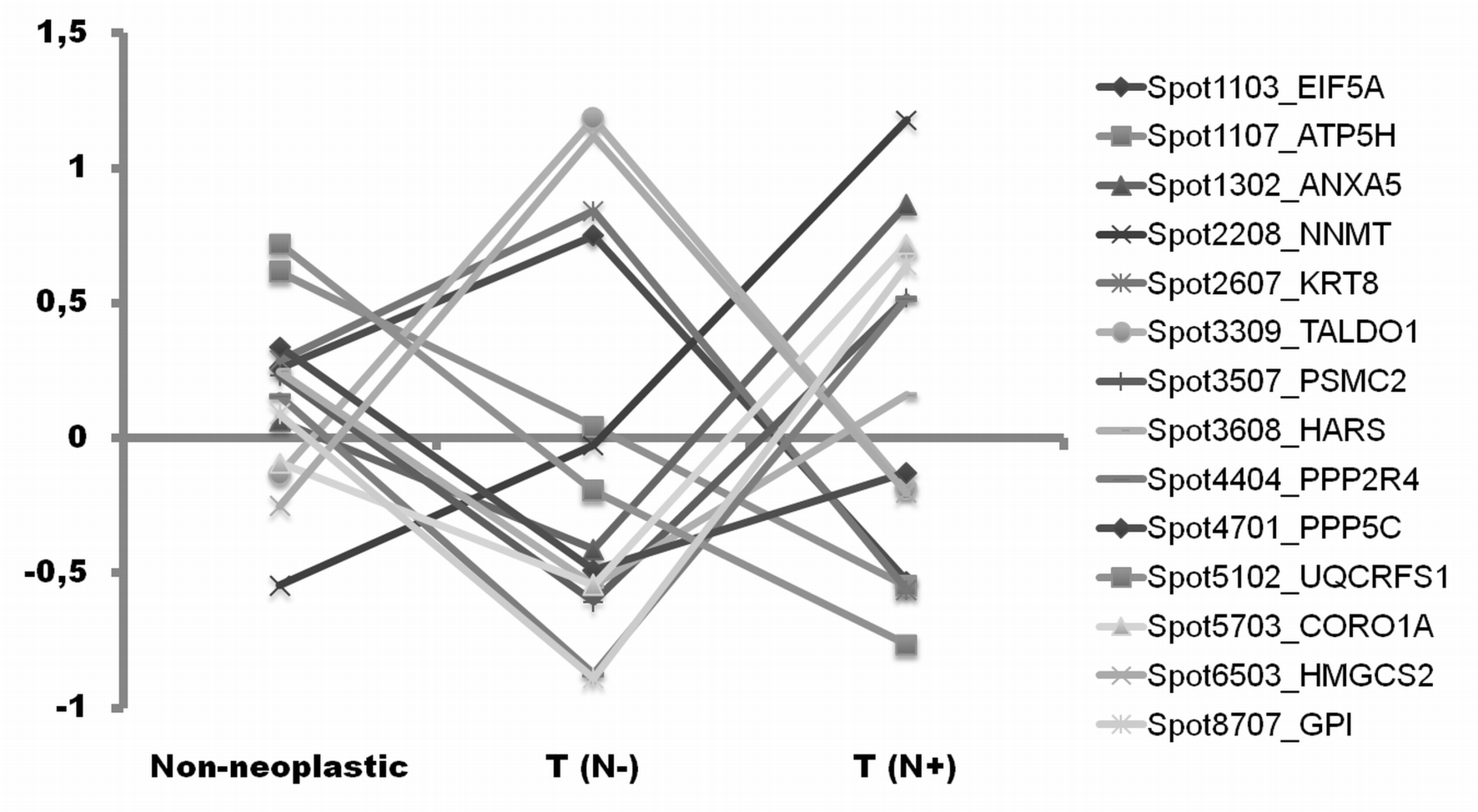

Supplement: Figure S1 — 14 significantly different proteins between tumors with and without lymph node metastasis. The normalized means (z-scores) of the expression in non-neoplastic, neoplastic without [T(N−)] and with lymph node metastasis [T(N+)] is presented. (TIF) [file pone.0042255.s001.tif]

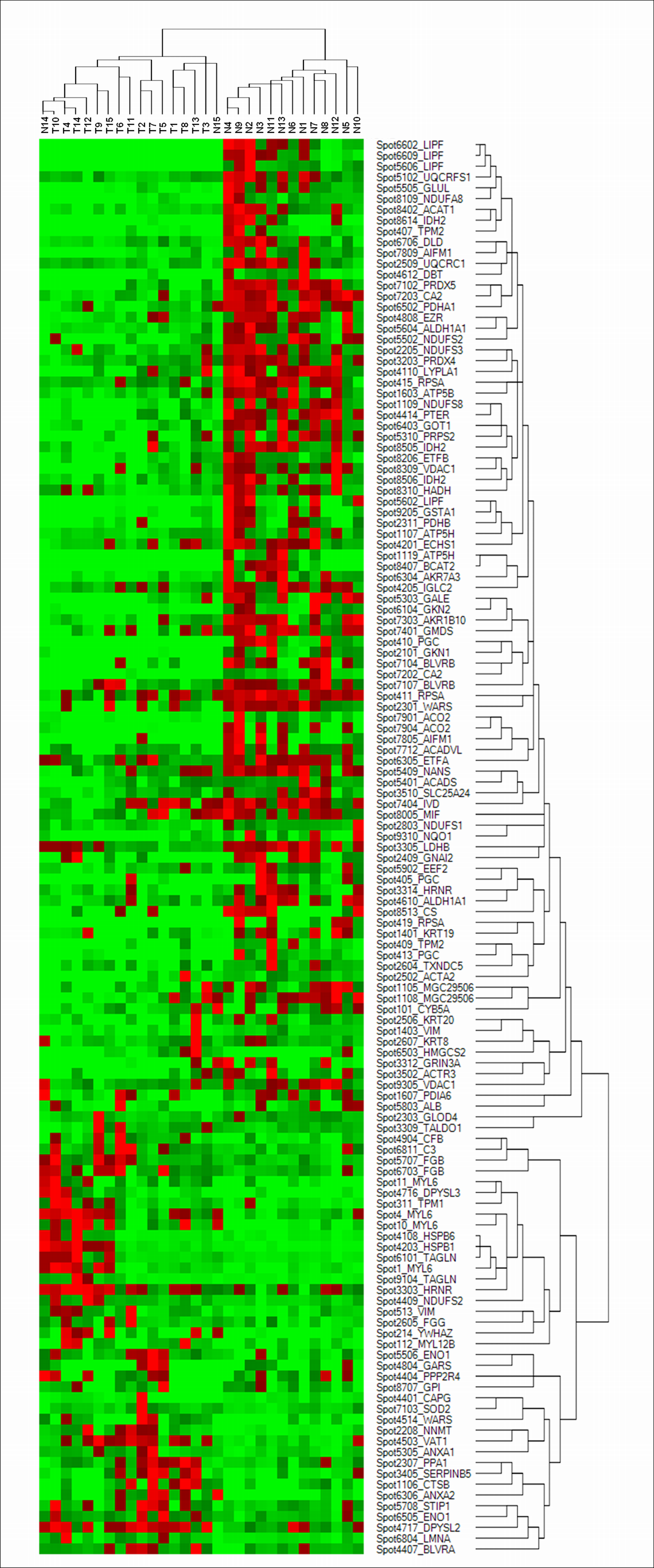

Supplement: Figure S2 — Protein-protein physical/functional interaction subnetworks in gastric carcinogenesis by generated Ingenuity Pathway Analysis tool. A) Cancer subnetwork revealed in the analysis of differentially expressed proteins between neoplastic and matched controls; B) Cellular assembly and organization, energy production, nucleic acid metabolism subnetwork revealed in the analysis of differentially expressed proteins between neoplastic and matched controls; C) Cellular assembly and organization subnetwork revealed in the analysis of differentially expressed proteins between controls and tumors with lymph node metastasis; D) Inflammatory subnetwork revealed in the analysis of differentially expressed proteins between controls and tumors without lymph node metastasis. Red: up-regulated proteins; Green: down-regulated proteins. (TIF) [file pone.0042255.s002.tif]

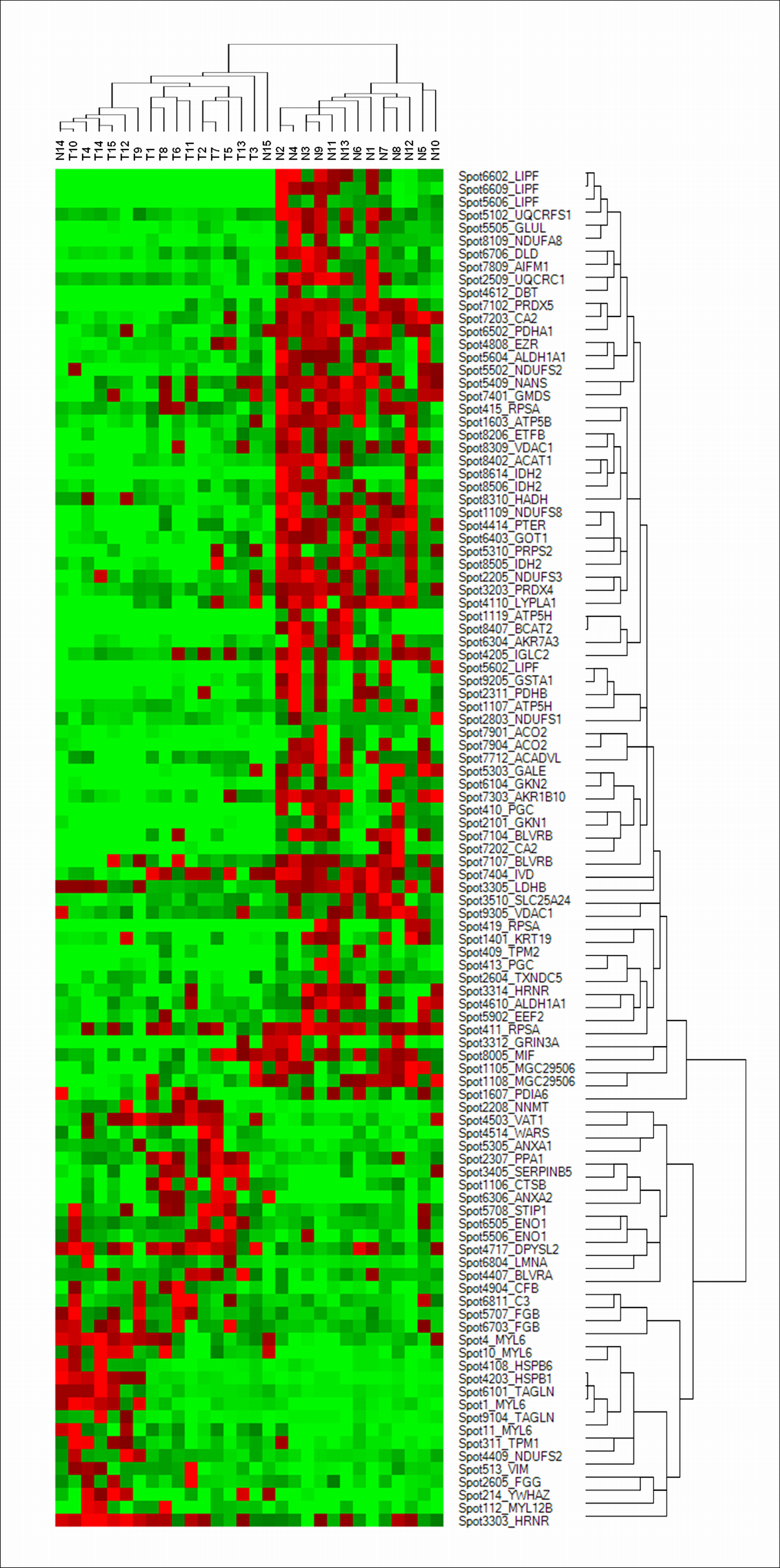

Supplement: Figure S3 — Differentially expressed by paired T-test analysis. Heat map represents the expression protein level. The samples are shown vertically and the proteins horizontally. Higher expressions are colored red, the lower ones in green. The dendrograms represent the distances between the clusters. (TIF) [file pone.0042255.s003.tif]

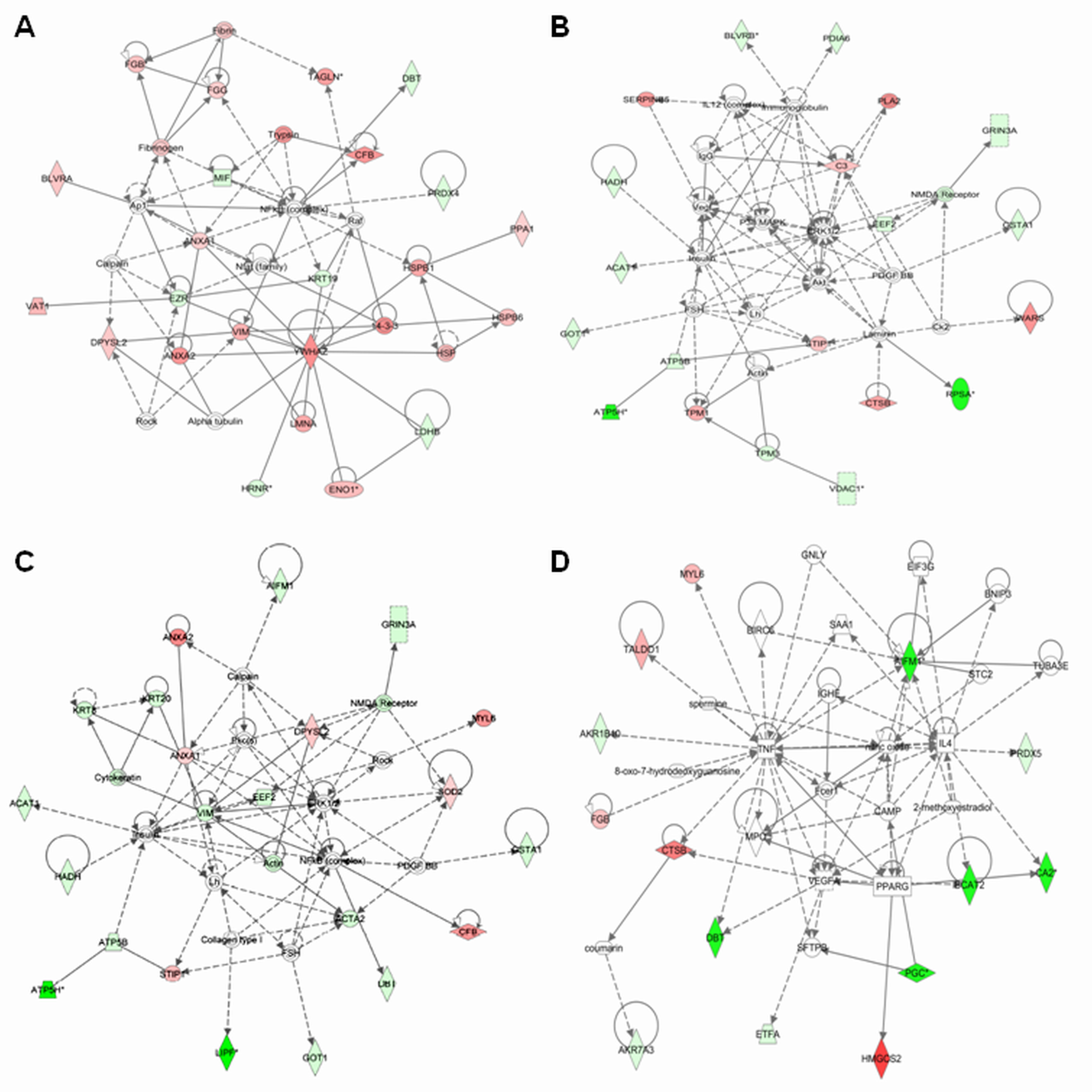

Supplement: Figure S4 — Differentially expressed by one-way ANOVA analysis. Heat map represents the expression protein level. The samples are shown vertically and the proteins horizontally. Higher expressions are colored red, the lower ones in green. The dendrograms represent the distances between the clusters. (TIF) [file pone.0042255.s004.tif]
