# Supplementary material for: Differential Proteomic Analysis of Noncardia Gastric Cancer from Individuals of Northern Brazil
Source: PLoS One. 2012 Jul 30;7(7):e42255. doi: 10.1371/journal.pone.0042255 (PMC3408468; doi:10.1371/journal.pone.0042255)
Supplement: Table S1 — Differentially expressed proteins between neoplastic and non-neoplastic gastric samples by paired T-test analysis. (DOCX) [file pone.0042255.s005.docx]

Table S1. Differentially expressed proteins between neoplastic and non-neoplastic gastric samples by paired T-test analysis.

| Spot | IPI # | Protein symbol | Description | Theor. MW (KDa) | Exp MW (KDa) | Theor. pI | Exp. pI | Mascot Score | Sequence coverage (%) | # matched peptides | Expression ratio (T/N) | p-value | Ref.^ǂ^ |
| --- | --- | --- | --- | --- | --- | --- | --- | --- | --- | --- | --- | --- | --- |
| 1 | IPI00335168 | MYL6 | Isoform Non-muscle of Myosin light polypeptide 6 | 17.09 | 16.84 | 4.56 | 3.84 | 327 | 29 | 10 | 3.659 | 0.031 | [1] |
| 4 | IPI00335168 | MYL6 | Isoform Non-muscle of Myosin light polypeptide 6 | 17.09 | 16.19 | 4.56 | 4.09 | 296 | 29 | 9 | 1.986 | 0.008 | [1] |
| 10 | IPI00335168 | MYL6 | Isoform Non-muscle of Myosin light polypeptide 6 | 17.09 | 16.52 | 4.56 | 3.8 | 312 | 36 | 10 | 3.501 | 0.004 | [1] |
| 11 | IPI00335168 | MYL6 | Isoform Non-muscle of Myosin light polypeptide 6 | 17.09 | 16.22 | 4.56 | 4.29 | 322 | 36 | 9 | 3.196 | 0.037 | [1] |
| 112 | IPI00033494 | MYL12B | Myosin regulatory light chain 12B | 19.82 | 18.24 | 4.71 | 4.16 | 182 | 57 | 9 | 4.799 | 0.034 |  |
| 214 | IPI00021263 | YWHAZ | 14-3-3 protein zeta/delta | 27.90 | 23.23 | 4.73 | 4.19 | 364 | 39 | 9 | 3.783 | 0.040 | [2] |
| 311 | IPI00000230 | TPM1 | Tropomyosin alpha-1 chain isoform 2 | 32.72 | 34.43 | 4.70 | 4.33 | 1294 | 61 | 34 | 2.645 | 0.048 | [3,4,5] |
| 409 | IPI00183968 | TPM3 | Tropomyosin alpha-3 chain isoform 1 | 32.99 | 36.95 | 4.70 | 4.22 | 359 | 22 | 12 | 0.179 | 0.026 | [4] |
| 410 | IPI00022213 | PGC | Gastricsin | 42.80 | 36.99 | 4.36 | 4.37 | 120 | 5 | 5 | * | 0.007 | [6] |
| 411 | IPI00413108 | RPSA | 33 kDa protein | 33.46 | 40.51 | 4.79 | 4.4 | 453 | 34 | 13 | 0.666 | 0.012 | [7] |
| 413 | IPI00022213 | PGC | Gastricsin | 42.80 | 36.97 | 4.36 | 4.55 | 92 | 5 | 2 | 0.094 | 0.048 | [6] |
| 415 | IPI00413108 | RPSA | 33 kDa protein | 33.46 | 40.2 | 4.79 | 4.66 | 527 | 43 | 14 | 0.587 | 0.016 | [7] |
| 419 | IPI00411639 | RPSA | Laminin receptor-like protein LAMRL5 | 33.09 | 39.11 | 4.84 | 4.69 | 50 | 26 | 5 | * | 0.033 | [7] |
| 513 | IPI00418471 | VIM | Vimentin | 53.68 | 48.46 | 5.06 | 4.56 | 399 | 28 | 16 | 2.604 | 0.040 | [7] |
| 1105 | IPI00102821 | MGC29506 | Isoform 1 of Plasma cell-induced resident endoplasmic reticulum protein | 21.02 | 18.53 | 5.37 | 5.11 | 81 | 6 | 2 | 0.362 | 0.013 |  |
| 1106 | IPI00295741 | CTSB | Cathepsin B | 30.78 | 21.26 | 5.88 | 5.17 | 102 | 10 | 5 | 3.008 | 0.020 |  |
| 1107 | IPI00220487 | ATP5H | Isoform 1 of ATP synthase subunit d. mitochondrial | 18.54 | 20.29 | 5.21 | 5.2 | 184 | 18 | 6 | 0.565 | 0.011 |  |
| 1108 | IPI00102821 | MGC29506 | Isoform 1 of Plasma cell-induced resident endoplasmic reticulum protein | 21.02 | 18.56 | 5.37 | 5.23 | 209 | 42 | 8 | 0.410 | 0.008 |  |
| 1109 | IPI00010845 | NDUFS8 | NADH dehydrogenase [ubiquinone] iron-sulfur protein 8. mitochondrial | 24.20 | 20.82 | 6.00 | 5.06 | 61 | 9 | 2 | 0.139 | 0.002 | [2,5] |
| 1119 | IPI00220487 | ATP5H | Isoform 1 of ATP synthase subunit d. mitochondrial | 18.54 | 20.2 | 5.21 | 4.96 | 271 | 54 | 9 | * | 0.035 |  |
| 1401 | IPI00479145 | KRT19 | Keratin. type I cytoskeletal 19 | 44.08 | 41.17 | 5.04 | 4.83 | 1045 | 58 | 29 | 0.413 | 0.017 | [2] |
| 1603 | IPI00303476 | ATP5B | ATP synthase subunit beta. Mitochondrial | 56.53 | 50.66 | 5.26 | 4.92 | 1199 | 46 | 28 | 0.487 | 0.001 |  |
| 1607 | IPI00299571 | PDIA6 | Isoform 2 of Protein disulfide-isomerase A6 | 54.38 | 50.13 | 5.17 | 5.08 | 627 | 25 | 14 | 0.470 | 0.037 | [4] |
| 2008 | - |  |  |  | 14.44 |  | 5.25 |  |  |  | 2.113 | 0.045 |  |
| 2101 | IPI00749381 | GKN1 | Gastrokine-1 | 22.27 | 19.57 | 5.90 | 5.43 | 162 | 18 | 5 | 0.008 | 0.001 | [3,4,8] |
| 2205 | IPI00025796 | NDUFS3 | NADH dehydrogenase [ubiquinone] iron-sulfur protein 3. mitochondrial | 30.34 | 22.35 | 6.99 | 5.76 | 254 | 21 | 7 | 0.429 | 0.045 |  |
| 2208 | IPI00027681 | NNMT | Nicotinamide N-methyltransferase | 30.01 | 22.92 | 5.56 | 5.53 | 201 | 20 | 5 | 7.002 | 0.006 | [2,5,9] |
| 2307 | IPI00015018 | PPA1 | Inorganic pyrophosphatase | 33.10 | 28.83 | 5.54 | 5.72 | 293 | 39 | 14 | 1.512 | 0.013 | [9] |
| 2311 | IPI00003925 | PDHB | Isoform 1 of Pyruvate dehydrogenase E1 component subunit beta. mitochondrial | 39.55 | 31.56 | 6.20 | 5.63 | 188 | 18 | 5 | 0.230 | 0.013 | [3] |
| 2509 | IPI00013847 | UQCRC1 | Cytochrome b-c1 complex subunit 1. mitochondrial | 53.30 | 47.09 | 5.94 | 5.73 | 512 | 31 | 18 | 0.534 | 0.002 | [3] |
| 2604 | IPI00171438 | TXNDC5 | Thioredoxin domain-containing protein 5 | 48.28 | 49.71 | 5.63 | 5.59 | 272 | 31 | 15 | 0.504 | 0.032 | [3] |
| 2605 | IPI00021891 | FGG | Isoform Gamma-B of Fibrinogen gamma chain | 52.11 | 51.49 | 5.37 | 5.6 | 106 | 10 | 9 | 1.671 | 0.048 | [10] |
| 2709 | IPI00219005 | FKBP4 | Peptidyl-prolyl cis-trans isomerase FKBP4 | 52.06 | 56.56 | 5.35 | 5.49 | 173 | L34 | 13 | 2.317 | 0.026 |  |
|  | IPI00013164 | PRPH | Peripherin | 53.73 |  | 5.37 |  | 163 | 35 | 17 |  |  |  |
|  | IPI00216049 | HNRNPK | Isoform 1 of Heterogeneous nuclear ribonucleoprotein K | 51.23 |  | 5.39 |  | 115 | 15 | 6 |  |  |  |
|  | IPI00010471 | LCP1 | Plastin-2 | 70.82 |  | 5.2 |  | 73 | 17 | 8 |  |  |  |
|  | IPI00418471 | VIM | Vimentin | 53.68 |  | 5.06 |  | 43 | 8 | 5 |  |  | [7] |
| 2803 | IPI00604664 | NDUFS1 | NADH-ubiquinone oxidoreductase 75 kDa subunit | 81.97 | 80.51 | 6.24 | 5.49 | 472 | 39 | 25 | 0.412 | 0.024 |  |
| 3103 | IPI00024919 | PRDX3 | Thioredoxin-dependent peroxide reductase. mitochondrial | 28.02 | 21.07 | 7.67 | 6.33 | 156 | 12 | 3 | 0.415 | 0.020 | [3] |
|  | IPI00002149 | SAR1B | GTP-binding protein SAR1b | 22.51 |  | 5.76 |  | 156 | 42 | 6 |  |  |  |
|  | IPI00219757 | GSTP1 | Glutathione S-transferase P | 23.57 |  | 5.43 |  | 149 | 28 | 5 |  |  | [7,9,11] |
| 3107 | IPI00291328 | NDUFV2 | NADH dehydrogenase [ubiquinone] flavoprotein 2. mitochondrial | 27.66 | 21.18 | 8.22 | 6.58 | 194 | 35 | 9 | 0.122 | 0.004 | [5] |
|  | IPI00334159 | VBP1 | von Hippel-Lindau binding protein 1. isoform CRA_b | 26.69 |  | 9.1 |  | 49 | 14 | 3 |  |  |  |
|  | IPI00219757 | GSTP1 | Glutathione S-transferase P | 23.57 |  | 5.43 |  | 49 | 19 | 3 |  |  | [7,9,11] |
| 3203 | IPI00011937 | PRDX4 | Peroxiredoxin-4 | 30.75 | 22.32 | 5.86 | 5.95 | 333 | 27 | 11 | 0.488 | 0.003 | [2] |
| 3206 | - |  |  |  | 22.7 |  | 6.32 |  |  |  | 0.644 | 0.024 |  |
| 3303 | IPI00398625 | HRNR | Hornerin | 28.31 | 30.51 | 10.05 | 5.88 | 237 | 2 | 6 | 1.548 | 0.005 |  |
| 3305 | IPI00219217 | LDHB | L-lactate dehydrogenase B chain | 36.90 | 31.09 | 5.71 | 6.18 | 664 | 40 | 20 | 0.653 | 0.005 | [3] |
| 3312 | IPI00455154 | GRIN3A | Glutamate [NMDA] receptor subunit 3A | 12.54 | 27.83 | 7.4 | 5.86 | 30 | 0 | 1 | 0.119 | 0.017 |  |
| 3314 | IPI00398625 | HRNR | Hornerin | 28.31 | 31.79 | 10.05 | 5.92 | 101 | 2 | 4 | 0.270 | 0.010 |  |
| 3405 | IPI00783625 | SERPINB5 | Isoform 1 of Serpin B5 | 42.53 | 38.35 | 5.72 | 6.46 | 726 | 49 | 23 | 3.198 | 0.015 |  |
| 3510 | IPI00337494 | SLC25A24 | Isoform 1 of Calcium-binding mitochondrial carrier protein SCaMC-1 | 53.55 | 43.53 | 6 | 6.66 | 254 | 36 | 17 | 0.447 | 0.002 |  |
| 4108 | IPI00022433 | HSPB6 | Heat shock protein beta-6 | 18.89 | 18.07 | 7.90 | 6.76 | 195 | 41 | 7 | 2.154 | 0.026 |  |
| 4110 | IPI00398727 | LYPLA1 | Isoform 2 of Acyl-protein thioesterase 1 | 23.20 | 20.96 | 6.05 | 6.88 | 79 | 14 | 3 | 0.433 | 0.001 |  |
| 4203 | IPI00025512 | HSPB1 | Heat shock protein beta-1 | 22.83 | 22.05 | 5.98 | 6.73 | 629 | 55 | 19 | 2.887 | 0.011 | [1,3,5,8,9,10,11,12] |
| 4205 | IPI00154742 | IGLC2 | IGL protein | 25.12 | 22.62 | 5.93 | 6.78 | 106 | 18 | 6 | 0.603 | 0.029 |  |
| 4301 | - |  |  |  |  |  |  |  |  |  | 1.793 | 0.044 |  |
| 4306 | IPI00186008 | STARD10 | PCTP-like protein | 33.43 | 30.92 | 6.67 | 6.79 | 80 | 12 | 3 | 0.148 | 0.003 |  |
|  | IPI00015809 | OSGEP | Probable O-sialoglycoprotein endopeptidase | 36.92 |  | 5.94 |  | 64 | 8 | 2 |  |  |  |
|  | IPI00219217 | LDHB | L-lactate dehydrogenase B chain | 36.90 |  | 5.71 |  | 64 | 17 | 5 |  |  | [3] |
| 4309 | IPI00793199 | ANXA4 | Annexin A4 | 36.29 | 26.38 | 5.84 | 6.79 | 312 | 33 | 10 | 0.487 | 0.025 |  |
|  | IPI00011416 | ECH1 | Delta(3.5)-Delta(2.4)-dienoyl-CoA isomerase. mitochondrial | 36.14 |  | 8.16 |  | 92 | 5 | 2 |  |  | [9] |
| 4407 | IPI00294158 | BLVRA | Biliverdin reductase A | 33.69 | 34.66 | 6.06 | 6.81 | 167 | 16 | 4 | 1.854 | 0.013 |  |
| 4409 | IPI00014177 | SEPT2 | Isoform 1 of Septin-2 | 41.69 | 41.29 | 6.15 | 6.84 | 112 | 8 | 4 | 2.130 | 0.042 | [5] |
| 4414 | IPI00100933 | PTER | Isoform 1 of Phosphotriesterase-related protein | 39.51 | 36.57 | 6.07 | 6.83 | 155 | 23 | 7 | 0.207 | 0.001 |  |
| 4501 | IPI00031461 | GDI2 | cDNA FLJ60299. highly similar to Rab GDP dissociation inhibitor beta | 51.58 | 48.16 | 8.37 | 6.73 | 206 | 21 | 9 | 1.779 | 0.007 |  |
|  | IPI00028091 | ACTR3 | Actin-related protein 3 | 47.80 |  | 5.61 |  | 74 | 14 | 5 |  |  |  |
| 4503 | IPI00156689 | VAT1 | Synaptic vesicle membrane protein VAT-1 homolog | 42.12 | 45.36 | 5.88 | 6.74 | 313 | 31 | 9 | 2.395 | 0.010 |  |
| 4514 | IPI00295400 | WARS | Isoform 1 of Tryptophanyl-tRNA synthetase. cytoplasmic | 53.47 | 47.07 | 5.83 | 6.76 | 259 | 20 | 8 | 3.580 | 0.033 | [3] |
| 4606 | IPI00218914 | ALDH1A1 | Retinal dehydrogenase 1 | 55.45 | 53.81 | 6.3 | 6.8 | 260 | 25 | 13 | 0.053 | 0.015 | [1] |
|  | IPI00180675 | TUBA1A | Tubulin alpha-1A chain | 50.79 |  | 4.94 |  | 85 | 19 | 6 |  |  | [7] |
|  | IPI00297779 | CCT2 | T-complex protein 1 subunit beta | 57.79 |  | 6.01 |  | 81 | 11 | 6 |  |  |  |
| 4608 | IPI00026516 | OXCT1 | Succinyl-CoA:3-ketoacid-coenzyme A transferase 1. mitochondrial | 56.58 | 54.98 | 7.14 | 6.83 | 511 | 46 | 17 | 0.452 | 0.013 | [3] |
|  | IPI00218343 | TUBA1C | Tubulin alpha-1C chain | 50.55 |  | 4.96 |  | 310 | 28 | 11 |  |  | [7] |
| 4610 | IPI00218914 | ALDH1A1 | Retinal dehydrogenase 1 | 55.45 | 54.11 | 6.30 | 6.86 | 334 | 22 | 8 | 0.388 | 0.019 | [1] |
| 4612 | IPI00003944 | DBT | Lipoamide acyltransferase component of branched-chain alpha-keto acid dehydrogenase complex. mitochondrial | 53.85 | 50.44 | 8.71 | 6.88 | 257 | 35 | 17 | 0.055 | 0.034 | [3] |
| 4717 | IPI00257508 | DPYSL2 | Dihydropyrimidinase-related protein 2 | 62.71 | 60.56 | 5.95 | 6.83 | 320 | 21 | 12 | 1.790 | 0.011 |  |
| 4808 | IPI00843975 | EZR | Ezrin | 69.48 | 84.02 | 5.94 | 6.81 | 821 | 34 | 6 | 0.541 | 0.005 | [7] |
| 4904 | IPI00921523 | CFB | Complement factor B | 86.85 | 119.81 | 6.67 | 6.83 | 144 | 15 | 12 | 3.502 | 0.018 |  |
| 5102 | IPI00026964 | UQCRFS1 | Cytochrome b-c1 complex subunit Rieske. Mitochondrial | 29.93 | 21.55 | 8.55 | 6.95 | 427 | 27 | 13 | 0.399 | 0.002 |  |
| 5303 | IPI00553131 | GALE | UDP-glucose 4-epimerase | 38.66 | 32.61 | 6.26 | 6.9 | 264 | 18 | 6 | 0.183 | 0.001 | [3] |
| 5305 | IPI00218918 | ANXA1 | Annexin A1 | 38.92 | 32.75 | 6.57 | 6.93 | 642 | 38 | 20 | 1.586 | 0.048 | [3,5] |
| 5310 | IPI00219617 | PRPS2 | Isoform 1 of Ribose-phosphate pyrophosphokinase 2 | 35.15 | 28.5 | 6.15 | 6.92 | 278 | 37 | 12 | 0.256 | 0.005 |  |
| 5409 | IPI00147874 | NANS | Sialic acid synthase | 40.74 | 37.82 | 6.29 | 7.1 | 423 | 35 | 13 | 0.541 | 0.016 | [3] |
| 5502 | IPI00025239 | NDUFS2 | NADH dehydrogenase [ubiquinone] iron-sulfur protein 2. mitochondrial | 52.91 | 44.87 | 7.21 | 6.92 | 199 | 14 | 8 | 0.244 | 0.008 |  |
| 5505 | IPI00010130 | GLUL | Glutamine synthetase | 42.67 | 42.83 | 6.43 | 6.95 | 386 | 26 | 12 | 0.102 | 0.002 |  |
| 5506 | IPI00465248 | ENO1 | Isoform alpha-enolase of Alpha-enolase | 47.48 | 48.17 | 7.01 | 7.05 | 708 | 30 | 17 | 2.249 | 0.008 | [1,3,4,7,11] |
| 5602 | IPI00009893 | LIPF | cDNA FLJ55598. highly similar to Gastric triacylglycerol lipase | 46.50 | 50.6 | 8.21 | 6.9 | 161 | 17 | 7 | 0.025 | 0.016 | [3] |
| 5604 | IPI00218914 | ALDH1A1 | Retinal dehydrogenase 1 | 55.45 | 53.6 | 6.30 | 6.93 | 918 | 40 | 21 | 0.389 | 0.004 | [1] |
| 5606 | IPI00009893 | LIPF | cDNA FLJ55598. highly similar to Gastric triacylglycerol lipase | 46.50 | 50.14 | 8.21 | 7 | 233 | 17 | 10 | 0.003 | 0.002 | [3] |
| 5609 | IPI00218914 | ALDH1A1 | Retinal dehydrogenase 1 | 55.45 | 54.48 | 6.30 | 7.12 | 133 | 9 | 4 | 0.473 | 0.022 | [1] |
|  | IPI00414320 | ANXA11 | cDNA FLJ55482. highly similar to Annexin A11 | 66.13 |  | 8.52 |  | 120 | 6 | 4 |  |  |  |
| 5610 | IPI00019376 | SEPT11 | Isoform 2 of Septin-11 | 51.08 | 52.35 | 6.54 | 6.96 | 447 | 32 | 14 | 17.542 | 0.040 |  |
|  | IPI00399007 | IGHG2 | Putative uncharacterized protein DKFZp686I04196 (Fragment) | 46.72 |  | 7.63 |  | 109 | 5 | 2 |  |  |  |
| 5707 | IPI00298497 | FGB | Fibrinogen beta chain | 56.58 | 56.7 | 8.54 | 6.95 | 220 | 13 | 7 | 2.057 | 0.016 | [13] |
| 5708 | IPI00013894 | STIP1 | Stress-induced-phosphoprotein 1 | 63.23 | 61.52 | 6.40 | 6.96 | 377 | 21 | 13 | 1.986 | 0.016 |  |
| 5716 | IPI00298497 | FGB | Fibrinogen beta chain | 56.58 | 56.27 | 8.54 | 6.88 | 195 | 28 | 12 | 11.387 | 0.034 | [13] |
|  | IPI00006713 | DNAJC3 | DnaJ homolog subfamily C member 3 | 58.00 |  | 5.83 |  | 44 | 3 | 2 |  |  |  |
| 5801 | IPI00219365 | MSN | Moesin | 67.89 | 74.51 | 6.08 | 6.89 | 570 | 41 | 32 | 1.727 | 0.029 |  |
|  | IPI00021405 | LMNA | Isoform A of Lamin-A/C | 74.38 |  | 6.57 |  | 206 | 20 | 13 |  |  |  |
|  | IPI00384282 | EZR | Cytovillin 2 (Fragment) | 16.29 |  | 9.32 |  | 99 | 33 | 10 |  |  | [7] |
| 5902 | IPI00186290 | EEF2 | Elongation factor 2 | 96.25 | 114.33 | 6.41 | 6.99 | 372 | 36 | 37 | 0.465 | 0.025 | [3,7] |
| 6101 | IPI00216138 | TAGLN | Transgelin | 22.65 | 20.59 | 8.87 | 7.16 | 555 | 41 | 15 | 2.469 | 0.007 | [1,5,8,12] |
| 6104 | IPI00329266 | GKN2 | Gastrokine-2 | 20.76 | 18.37 | 7.03 | 7.58 | 201 | 26 | 7 | 0.034 | 0.002 |  |
| 6304 | IPI00293721 | AKR7A3 | Aflatoxin B1 aldehyde reductase member 3 | 37.58 | 32.61 | 6.67 | 7.32 | 351 | 33 | 11 | 0.144 | 0.002 | [3] |
| 6306 | IPI00418169 | ANXA2 | Isoform 2 of Annexin A2 | 40.67 | 31.93 | 8.53 | 7.45 | 428 | 36 | 12 | 3.543 | 0.046 | [7,11] |
| 6308 | IPI00015262 | CNN2 | Calponin-2 | 34.07 | 30.31 | 6.95 | 7.31 | 91 | 23 | 6 | 9.267 | 0.008 |  |
|  | IPI00916111 | MDH1 | Malate dehydrogenase | 38.92 |  | 8.11 |  | 81 | 15 | 5 |  |  | [3,7] |
| 6403 | IPI00219029 | GOT1 | Aspartate aminotransferase. cytoplasmic | 46.45 | 40.63 | 6.52 | 7.44 | 379 | 26 | 9 | 0.220 | 0.001 | [3] |
| 6502 | IPI00922697 | PDHA1 | Pyruvate dehydrogenase E1 component subunit alpha. somatic form. mitochondrial | 43.95 | 43.32 | 8.35 | 7.23 | 315 | 22 | 9 | 0.281 | 0.001 |  |
| 6505 | IPI00465248 | ENO1 | Isoform alpha-enolase of Alpha-enolase | 47.48 | 47.95 | 7.01 | 7.41 | 1449 | 53 | 29 | 1.762 | 0.002 | [1,3,4,7,11] |
| 6602 | IPI00009893 | LIPF | cDNA FLJ55598. highly similar to Gastric triacylglycerol lipase | 46.50 | 50.01 | 8.21 | 7.26 | 249 | 17 | 10 | * | 0.001 | [3] |
| 6609 | IPI00009893 | LIPF | cDNA FLJ55598. highly similar to Gastric triacylglycerol lipase | 46.50 | 50 | 8.21 | 7.56 | 268 | 17 | 12 | * | 0.002 | [3] |
| 6703 | IPI00298497 | FGB | Fibrinogen beta chain | 56.58 | 56.66 | 8.54 | 7.2 | 382 | 37 | 17 | 1.741 | 0.022 | [13] |
| 6704 | IPI00007074 | YARS | Tyrosyl-tRNA synthetase. cytoplasmic | 59.45 | 58.81 | 6.61 | 7.25 | 561 | 57 | 33 | 0.643 | 0.036 |  |
|  | IPI00465436 | CAT | Catalase | 59.95 |  | 6.9 |  | 33 | 4 | 2 |  |  | [7] |
| 6706 | IPI00015911 | DLD | Dihydrolipoyl dehydrogenase. mitochondrial | 54.71 | 56.12 | 7.95 | 7.32 | 412 | 25 | 10 | 0.601 | 0.026 | [6] |
| 6804 | IPI00216952 | LMNA | Isoform C of Prelamin-A/C | 65.15 | 77.03 | 6.40 | 7.25 | 600 | 29 | 16 | 2.822 | 0.012 |  |
| 6811 | IPI00942927 | C3 | cDNA FLJ57339. highly similar to Complement C3 | 12.34 | 71.23 | 6.50 | 7.58 | 748 | 28 | 25 | 1.996 | 0.039 |  |
| 7102 | IPI00024915 | PRDX5 | Isoform Mitochondrial of Peroxiredoxin-5. Mitochondrial | 22.30 | 17.08 | 8.93 | 7.65 | 120 | 17 | 3 | 0.169 | 0.001 | [8,10] |
| 7104 | IPI00783862 | BLVRB | Flavin reductase | 22.22 | 21.1 | 7.13 | 7.92 | 129 | 35 | 5 | 0.182 | 0.001 | [10] |
| 7107 | IPI00783862 | BLVRB | Flavin reductase | 22.22 | 21.11 | 7.13 | 8.21 | 327 | 38 | 8 | 0.535 | 0.036 | [10] |
| 7202 | IPI00218414 | CA2 | Carbonic anhydrase 2 | 29.29 | 24.13 | 6.87 | 7.66 | 386 | 43 | 15 | 0.024 | 0.039 | [1,4,7,11] |
| 7203 | IPI00218414 | CA2 | Carbonic anhydrase 2 | 29.29 | 22.88 | 6.87 | 7.67 | 427 | 47 | 16 | 0.339 | 0.001 | [1,4,7,11] |
| 7301 | - |  |  |  | 26.68 |  | 7.65 |  |  |  | 0.411 | 0.031 |  |
| 7303 | IPI00105407 | AKR1B10 | Aldo-keto reductase family 1 member B10 | 36.23 | 31.5 | 7.12 | 7.85 | 598 | 37 | 17 | 0.312 | 0.002 | [5] |
| 7401 | IPI00030207 | GMDS | GDP-mannose 4.6 dehydratase | 42.27 | 40.35 | 6.87 | 7.62 | 152 | 16 |  | 0.441 | 0.017 |  |
| 7402 | IPI00029561 | NDUFA10 | NADH dehydrogenase [ubiquinone] 1 alpha subcomplex subunit 10. mitochondrial | 41.07 | 37.4 | 8.67 | 7.67 | 89 | 13 | 4 | 0.168 | 0.006 | [6] |
|  | IPI00398625 | HRNR | Hornerin | 28.31 |  | 10.05 |  | 35 | 2 | 4 |  |  |  |
| 7404 | IPI00645805 | IVD | Isovaleryl-CoA dehydrogenase. mitochondrial | 46.80 | 41.38 | 8.45 | 7.73 | 107 | 9 |  | 0.654 | 0.027 |  |
| 7407 | IPI00030363 | ACAT1 | Acetyl-CoA acetyltransferase. mitochondrial | 45.46 | 40.95 | 8.89 | 8.08 | 235 | 41 | 15 | 0.254 | 0.006 |  |
|  | IPI00028888 | HNRNPD | Isoform 1 of Heterogeneous nuclear ribonucleoprotein D0 | 38.51 |  | 7.62 |  | 60 | 6 | 2 |  |  |  |
| 7508 | IPI00015141 | CKMT2 | Creatine kinase S-type. mitochondrial | 47.99 | 44.6 | 8.46 | 8.02 | 199 | 31 | 9 | 0.283 | 0.050 | [4] |
|  | IPI00028888 | HNRNPD | Isoform 1 of Heterogeneous nuclear ribonucleoprotein D0 | 38.58 |  | 7.62 |  | 72 | 10 | 4 |  |  |  |
|  | IPI00169383 | PGK1 | Phosphoglycerate kinase 1 | 44.99 |  | 8.3 |  | 39 | 4 | 2 |  |  | [7] |
| 7603 | IPI00440493 | ATP5A1 | ATP synthase subunit alpha. mitochondrial | 59.83 | 53.32 | 9.16 | 7.78 | 178 | 17 | 8 | 0.332 | 0.009 |  |
|  | IPI00384938 | IGHG1 | LOC100290146;LOC100294459 Putative uncharacterized protein DKFZp686N02209 | 53.50 |  | 8.74 |  | 90 | 14 | 6 |  |  |  |
|  | IPI00019888 | ALDH5A1 | Succinate-semialdehyde dehydrogenase. mitochondrial | 58.03 |  | 8.62 |  | 73 | 12 | 7 |  |  |  |
|  | IPI00399007 | IGHG2 | Putative uncharacterized protein DKFZp686I04196 (Fragment) | 46.72 |  | 7.63 |  | 45 | 10 | 4 |  |  |  |
| 7712 | IPI00028031 | ACADVL | cDNA FLJ56425. highly similar to Very-long-chain specific acyl-CoAdehydrogenase. mitochondrial | 75.62 | 61.91 | 9.16 | 8.24 | 602 | 33 | 25 | 0.462 | 0.018 |  |
| 7809 | IPI00000690 | AIFM1 | Isoform 1 of Apoptosis-inducing factor 1. mitochondrial | 67.14 | 63.19 | 9.04 | 8.13 | 497 | 25 | 14 | 0.131 | 0.007 | [3] |
| 7901 | IPI00017855 | ACO2 | Aconitate hydratase mitochondrial | 86.11 | 96.15 | 7.36 | 7.65 | 165 | 6 | 4 | 0.114 | 0.032 | [3,7] |
| 7904 | IPI00017855 | ACO2 | Aconitate hydratase. mitochondrial | 86.11 | 95.1 | 7.36 | 7.82 | 464 | 19 | 15 | 0.018 | 0.009 | [3,7] |
| 8005 | IPI00293276 | MIF | Macrophage migration inhibitory factor | 12.64 | 12.5 | 7.74 | 8.84 | 74 | 17 | 2 | 0.591 | 0.040 |  |
| 8109 | IPI00219034 | NDUFA8 | NADH dehydrogenase [ubiquinone] 1 alpha subcomplex subunit 8 | 20.55 | 18.79 | 7.57 | 8.53 | 112 | 31 | 6 | 0.095 | 0.009 |  |
| 8206 | IPI00004902 | ETFB | Isoform 1 of Electron transfer flavoprotein subunit beta | 28.05 | 23.35 | 8.24 | 8.96 | 422 | 44 | 12 | 0.298 | 0.012 | [11] |
| 8208 | IPI00215901 | AK2 | Isoform 1 of Adenylate kinase 2. mitochondrial | 26.69 | 23.63 | 7.67 | 8.52 | 161 | 56 | 11 | 0.156 | 0.001 |  |
|  | IPI00171199 | PSMA3 | Isoform 2 of Proteasome subunit alpha type-3 | 27.86 |  | 5.19 |  | 76 | 14 | 3 |  |  |  |
|  | IPI00465431 | LGALS3 | Galectin-3 | 26.19 |  | 8.57 |  | 38 | 13 | 3 |  |  | [7] |
| 8302 | IPI00219018 | GAPDH | Glyceraldehyde-3-phosphate dehydrogenase | 36.20 | 33.94 | 8.57 | 8.45 | 174 | 22 | 7 | 0.103 | 0.047 | [7] |
|  | IPI00029733 | AKR1C1 | Aldo-keto reductase family 1 member C1 | 37.22 |  | 8.02 |  | 166 | 23 | 7 |  |  |  |
| 8309 | IPI00216308 | VDAC1 | Voltage-dependent anion-selective channel protein 1 | 30.87 | 27.21 | 8.62 | 9.09 | 261 | 21 | 7 | 0.280 | 0.002 | [1,11] |
| 8310 | IPI00294398 | HADH | Isoform 1 of Hydroxyacyl-coenzyme A dehydrogenase. mitochondrial | 34.33 | 27.41 | 8.88 | 9.18 | 370 | 26 | 11 | 0.489 | 0.001 | [8] |
| 8402 | IPI00030363 | ACAT1 | Acetyl-CoA acetyltransferase. mitochondrial | 45.46 | 40.95 | 8.98 | 8.57 | 443 | 31 | 15 | 0.225 | 0.003 |  |
| 8404 | IPI00021439 | ACTB | Actin. cytoplasmic 1 | 42.05 | 38.22 | 5.29 | 8.85 | 51 | 14 | 3 | 0.091 | 0.002 | [3,7,11] |
|  | IPI00396258 | BCAT2 | Isoform A of Branched-chain-amino-acid aminotransferase. mitochondrial | 44.66 |  | 8.88 |  | 49 | 9 | 4 |  |  |  |
| 8407 | IPI00396258 | BCAT2 | Isoform A of Branched-chain-amino-acid aminotransferase. mitochondrial | 44.66 | 37.91 | 8.88 | 8.42 | 68 | 10 | 5 | * | 0.009 |  |
| 8501 | IPI00305383 | UQCRC2 | Cytochrome b-c1 complex subunit 2. mitochondrial | 48.58 | 44.24 | 8.74 | 8.34 | 220 | 17 | 13 | 0.222 | 0.002 |  |
|  | IPI00011107 | IDH2 | Isocitrate dehydrogenase [NADP]. Mitochondrial | 51.33 |  | 8.88 |  | 351 | 33 | 9 |  |  | [5] |
| 8505 | IPI00011107 | IDH2 | Isocitrate dehydrogenase [NADP]. mitochondrial | 51.33 | 44.66 | 8.88 | 8.66 | 584 | 27 | 22 | 0.316 | 0.010 | [5] |
| 8506 | IPI00011107 | IDH2 | Isocitrate dehydrogenase [NADP]. mitochondrial | 51.33 | 44.75 | 8.88 | 8.91 | 814 | 31 | 22 | 0.346 | 0.004 | [5] |
| 8606 | IPI00024990 | ALDH6A1 | Methylmalonate-semialdehyde dehydrogenase [acylating]. mitochondrial | 58.26 | 54.84 | 8.72 | 8.48 | 196 | 8 | 5 | * | 0.022 |  |
|  | IPI00440493 | ATP5A1 | ATP synthase subunit alpha. mitochondrial | 59.83 |  | 9.16 |  | 151 | 9 | 4 |  |  |  |
|  | IPI00384938 | IGHV4-31 | Putative uncharacterized protein DKFZp686N02209 | 53.50 |  | 8.74 |  | 61 | 6 | 2 |  |  |  |
| 8614 | IPI00011107 | IDH2 | Isocitrate dehydrogenase [NADP]. mitochondrial | 50.88 | 46.03 | 8.88 | 8.34 | 297 | 29 | 12 | 0.020 | 0.014 | [5] |
| 9104 | IPI00216138 | TAGLN | Transgelin | 22.65 | 21.1 | 8.87 | 9.48 | 779 | 61 | 20 | 3.187 | 0.049 | [1,5,8,12] |
| 9205 | IPI00657682 | GSTA1 | Glutathione S-transferase A1 | 25.67 | 22.06 | 8.91 | 10 | 238 | 27 | 9 | 0.145 | 0.016 | [7,9,11] |
| 9302 | IPI00396378 | HNRNPA2B1 | Isoform B1 of Heterogeneous nuclear ribonucleoproteins A2/B1 | 37.46 | 32.01 | 8.97 | 9.27 | 287 | 24 | 9 | 0.366 | 0.006 | [5,7,11] |
|  | IPI00291006 | MDH2 | Malate dehydrogenase. mitochondrial | 35.94 |  | 8.92 |  | 67 | 12 | 3 |  |  | [3,7] |
| 9305 | IPI00216308 | VDAC1 | Voltage-dependent anion-selective channel protein 1 | 30.87 | 27.13 | 8.62 | 9.4 | 523 | 43 | 12 | 0.605 | 0.004 | [1,11] |

* Absent in tumor samples. ^ǂ^ Previous proteomic studies of gastric tumor that identified the related protein as differentially expressed. In some cases, a previous study identified proteins without description of isoforms and these proteins were also included in the present list, e.g. some studies identified malate dehydrogenase and these were listed in the table as related to MDH1 and MDH2. T: tumor samples; N: non-neoplastic samples. Theor: Theoretical; Exp: Experimental.

1. Bai Z, Ye Y, Liang B, Xu F, Zhang H, et al. (2011) Proteomics-based identification of a group of apoptosis-related proteins and biomarkers in gastric cancer. Int J Oncol 38: 375-383.

2. Jang JS, Cho HY, Lee YJ, Ha WS, Kim HW (2004) The differential proteome profile of stomach cancer: identification of the biomarker candidates. Oncol Res 14: 491-499.

3. Cai Z, Zhao JS, Li JJ, Peng DN, Wang XY, et al. (2010) A combined proteomics and metabolomics profiling of gastric cardia cancer reveals characteristic dysregulations in glucose metabolism. Mol Cell Proteomics 9: 2617-2628.

4. He QY, Cheung YH, Leung SY, Yuen ST, Chu KM, et al. (2004) Diverse proteomic alterations in gastric adenocarcinoma. Proteomics 4: 3276-3287.

5. Zhang J, Kang B, Tan X, Bai Z, Liang Y, et al. (2007) Comparative analysis of the protein profiles from primary gastric tumors and their adjacent regions: MAWBP could be a new protein candidate involved in gastric cancer. J Proteome Res 6: 4423-4432.

6. Ebert MP, Rocken C (2006) Molecular screening of gastric cancer by proteome analysis. Eur J Gastroenterol Hepatol 18: 847-853.

7. Yoshihara T, Kadota Y, Yoshimura Y, Tatano Y, Takeuchi N, et al. (2006) Proteomic alteration in gastic adenocarcinomas from Japanese patients. Mol Cancer 5: 75.

8. Li N, Zhang J, Liang Y, Shao J, Peng F, et al. (2007) A controversial tumor marker: is SM22 a proper biomarker for gastric cancer cells? J Proteome Res 6: 3304-3312.

9. Nishigaki R, Osaki M, Hiratsuka M, Toda T, Murakami K, et al. (2005) Proteomic identification of differentially-expressed genes in human gastric carcinomas. Proteomics 5: 3205-3213.

10. Wu C, Luo Z, Chen X, Wu C, Yao D, et al. (2009) Two-dimensional differential in-gel electrophoresis for identification of gastric cancer-specific protein markers. Oncol Rep 21: 1429-1437.

11. Cheng Y, Zhang J, Li Y, Wang Y, Gong J (2007) Proteome analysis of human gastric cardia adenocarcinoma by laser capture microdissection. BMC Cancer 7: 191.

12. Ryu JW, Kim HJ, Lee YS, Myong NH, Hwang CH, et al. (2003) The proteomics approach to find biomarkers in gastric cancer. J Korean Med Sci 18: 505-509.

13. Wang KJ, Wang RT, Zhang JZ (2004) Identification of tumor markers using two-dimensional electrophoresis in gastric carcinoma. World J Gastroenterol 10: 2179-2183.
