# Supplementary material for: Differential Proteomic Analysis of Noncardia Gastric Cancer from Individuals of Northern Brazil
Source: PLoS One. 2012 Jul 30;7(7):e42255. doi: 10.1371/journal.pone.0042255 (PMC3408468; doi:10.1371/journal.pone.0042255)
Supplement: Table S2 — Differentially expressed proteins among non-neoplastic gastric samples, neoplastic without lymph node metastasis and neoplastic with lymph node metastasis by ANOVA one-way analysis. (DOCX) [file pone.0042255.s006.docx]

Table S2. Differentially expressed proteins among non-neoplastic gastric samples, neoplastic without lymph node metastasis and neoplastic with lymph node metastasis by ANOVA one-way analysis.

| Spot | IPI # | Protein symbol | Description | Theor. MW (KDa) | Exp. MW (KDa) | Theor. pI | Exp. pI | Mascot Score | Sequence coverage (%) | # matched peptides | Tissue | Mean ratio | p-value | Ref.^ǂ^ |
| --- | --- | --- | --- | --- | --- | --- | --- | --- | --- | --- | --- | --- | --- | --- |
| 4^†^ | IPI00335168 | MYL6 | Isoform Non-muscle of Myosin light polypeptide 6 | 17.09 | 16.19 | 4.56 | 4.09 | 296 | 29 | 9.00 | T(N-)>No | 2.093 | 0.013 | [1] |
| 11^†^ | IPI00335168 | MYL6 | Isoform Non-muscle of Myosin light polypeptide 6 | 17.09 | 16.22 | 4.56 | 4.29 | 322 | 36 | 9.00 | T(N+)>No | 3.644 | 0.032 | [1] |
| 101 | IPI00182933 | CYB5A | Isoform 2 of Cytochrome b5 | 11.26 | 17.52 | 5.02 | 4.19 | 188 | 64 | 7.00 | T(N+)<No | 0.354 | 0.031 |  |
| 112^†^ | IPI00033494 | MYL12B | Myosin regulatory light chain 12B | 19.82 | 18.24 | 4.71 | 4.16 | 182 | 57 | 9.00 | T(N-)>No | 6.149 | 0.026 |  |
| 305 | - |  |  |  | 29.36 |  | 4.63 |  |  |  | T(N-)<No | * | 0.023 |  |
| 405 | IPI00022213 | PGC | Gastricsin | 42.80 | 37.64 | 4.36 | 3.9 | 118 | 6 | 4.00 | T(N-)<No | * | 0.023 | [2] |
| 407 | IPI00220709 | TPM2 | Isoform 2 of Tropomyosin beta chain | 33.03 | 36.94 | 4.63 | 4.13 | 514 | 34 | 14.00 | T(N-)<No | 0.061 | 0.033 | [1,3,4] |
| 410^†^ | IPI00022213 | PGC | Gastricsin | 42.80 | 36.99 | 4.36 | 4.37 | 120 | 5 | 5.00 | T(N-)<No | * | 0.003 | [2] |
|  |  |  |  |  |  |  |  |  |  |  | T(N+)<No | ** | 0.003 |  |
| 411^†^ | IPI00413108 | RPSA | 33 kDa protein | 33.46 | 40.51 | 4.79 | 4.4 | 453 | 34 | 13.00 | T(N+)<No | 0.642 | 0.013 | [5] |
| 413^†^ | IPI00022213 | PGC | Gastricsin | 42.80 | 36.97 | 4.36 | 4.55 | 92 | 5 | 2.00 | T(N-)<No | 0.152 | 0.048 | [2] |
|  |  |  |  |  |  |  |  |  |  |  | T(N+)<No | 0.065 | 0.023 |  |
| 419^†^ | IPI00411639 | RPSA | Laminin receptor-like protein LAMRL5 | 33.09 | 39.11 | 4.84 | 4.69 | 50 | 26 | 5.00 | T(N-)<No | * | 0.038 | [5] |
|  |  |  |  |  |  |  |  |  |  |  | T(N+)<No | ** | 0.038 |  |
| 1103 | IPI00376005 | EIF5A | Isoform 2 of Eukaryotic translation initiation factor 5A-1 | 20.44 | 17.59 | 6.52 | 5.03 | 211 | 27 | 8.00 | T(N+)<T(N-) | 0.349 | 0.026 |  |
| 1105^†^ | IPI00102821 | MGC29506 | Isoform 1 of Plasma cell-induced resident endoplasmic reticulum protein | 21.02 | 18.53 | 5.37 | 5.11 | 81 | 6 | 2.00 | T(N+)<No | 0.283 | 0.032 |  |
| 1106^†^ | IPI00295741 | CTSB | Cathepsin B | 30.78 | 21.26 | 5.88 | 5.17 | 102 | 10 | 4.00 | T(N-)>No | 3.822 | 0.039 |  |
| 1107^†^ | IPI00220487 | ATP5H | Isoform 1 of ATP synthase subunit d. mitochondrial | 18.54 | 20.29 | 5.21 | 5.2 | 184 | 18 | 6.00 | T(N+)<No | 0.482 | 0.002 |  |
|  |  |  |  |  |  |  |  |  |  |  | T(N+)<T(N-) | 0.622 | 0.004 |  |
| 1108^†^ | IPI00102821 | MGC29506 | Isoform 1 of Plasma cell-induced resident endoplasmic reticulum protein | 21.02 | 18.56 | 5.37 | 5.23 | 209 | 42 | 8.00 | T(N+)<No | 0.231 | 0.003 |  |
| 1109^†^ | IPI00010845 | NDUFS8 | NADH dehydrogenase [ubiquinone] iron-sulfur protein 8. mitochondrial | 24.20 | 20.82 | 6.00 | 5.06 | 61 | 9 | 2.00 | T(N-)<No | 0.197 | 0.003 | [6,7] |
|  |  |  |  |  |  |  |  |  |  |  | T(N+)<No | 0.110 | 0.000 |  |
| 1112^†^ | IPI00005537 | MRPL12 | cDNA FLJ60124. highly similar to Mitochondrial dicarboxylate carrier | 48.58 | 18.7 | 9.58 | 5.06 | 113 | 14 | 5.00 | T(N+)<T(N-) | 0.319 | 0.035 |  |
|  | IPI00023004 | EIF1AY | EIF1AY Eukaryotic translation initiation factor 1A. Y-chromosomal | 16.55 |  | 5.07 |  | 89 | 18 | 4.00 |  |  |  |  |
| 1119 | IPI00220487 | ATP5H | Isoform 1 of ATP synthase subunit d. mitochondrial | 18.54 | 20.2 | 5.21 | 4.96 | 271 | 54 | 9.00 | T(N-)<No | * | 0.027 |  |
|  |  |  |  |  |  |  |  |  |  |  | T(N+)<No | ** | 0.027 |  |
| 1302 | IPI00329801 | ANXA5 | Annexin A5 | 35.97 | 27.11 | 4.94 | 4.84 | 727 | 38 | 23.00 | T(N+)<T(N-) | 1.760 | 0.002 | [8,9] |
| 1305 | IPI00025465 | OGN | cDNA FLJ59205. highly similar to Mimecan | 40.87 | 29.65 | 8.08 | 4.99 | 86 | 14 | 4.00 | T(N+)<No | 0.249 | 0.018 |  |
|  | IPI00470631 | COQ9 | Isoform 1 of Ubiquinone biosynthesis protein COQ9. mitochondrial | 35.66 |  | 5.61 |  | 74 | 19 | 5.00 |  |  |  |  |
| 1403 | IPI00418471 | VIM | Vimentin | 53.62 | 40.95 | 5.06 | 5.01 | 237 | 9 | 4.00 | T(N+)<No | 0.389 | 0.015 | [5] |
| 1507 | - |  |  |  | 45.75 |  | 5.18 |  |  |  | T(N-)<No | 0.266 | 0.046 |  |
|  |  |  |  |  |  |  |  |  |  |  | T(N+)<No | 0.209 | 0.011 |  |
| 1603^†^ | IPI00303476 | ATP5B | ATP synthase subunit beta. mitochondrial | 56.53 | 50.66 | 5.26 | 4.92 | 1199 | 46 | 28.00 | T(N+)<No | 0.441 | 0.001 |  |
| 2101^†^ | IPI00749381 | GKN1 | Gastrokine-1 | 22.27 | 19.57 | 5.90 | 5.43 | 162 | 18 | 5.00 | T(N-)<No | * | 0.001 | [3,4,10] |
|  |  |  |  |  |  |  |  |  |  |  | T(N+)<No | 0.012 | 0.001 |  |
| 2205^†^ | IPI00025796 | NDUFS3 | NADH dehydrogenase [ubiquinone] iron-sulfur protein 3. Mitocondrial | 30.34 | 22.35 | 6.99 | 5.76 | 254 | 21 | 7.00 | T(N+)<No | 0.344 | 0.007 |  |
| 2208^†^ | IPI00027681 | NNMT | Nicotinamide N-methyltransferase | 30.01 | 22.92 | 5.56 | 5.53 | 201 | 20 | 5.00 | T(N+)>No | 8.748 | 0.005 | [6,7,8] |
|  |  |  |  |  |  |  |  |  |  |  | T(N+)>T(N-) | 2.658 | 0.046 |  |
| 2301 | IPI00295400 | WARS | Tryptophanyl-tRNA synthetase. cytoplasmic | 53.13 | 32.03 | 5.83 | 5.43 | 32 | 1 | 1.00 | T(N+)<No | 0.354 | 0.020 | [10] |
| 2303 | IPI00032575 | GLOD4 | Isoform 1 of Glyoxalase domain-containing protein 4 | 35.17 | 27.24 | 5.40 | 5.56 | 217 | 24 | 8.00 | T(N+)<No | 0.635 | 0.006 |  |
| 2307^†^ | IPI00015018 | PPA1 | Inorganic pyrophosphatase | 33.10 | 28.83 | 5.54 | 5.72 | 293 | 39 | 14.00 | T(N+)>No | 1.504 | 0.047 | [8] |
| 2310 | - |  |  |  | 35.3 |  | 5.42 |  |  |  | T(N+)<No | 0.368 | 0.024 |  |
| 2311^†^ | IPI00003925 | PDHB | Isoform 1 of Pyruvate dehydrogenase E1 component subunit beta. mitochondrial | 39.55 | 31.56 | 6.20 | 5.63 | 188 | 18 | 5.00 | T(N-)<No | 0.176 | 0.023 | [10] |
| 2409 | IPI00220578 | GNAI3 | Guanine nucleotide-binding protein G(k) subunit alpha | 41.08 | 38.17 | 5.5 | 5.63 | 65 | 7 | 2.00 | T(N-)<No | 0.174 | 0.047 | [5] |
| 2502 | IPI00008603 | ACTA2 | Actin. aortic smooth muscle | 42.38 | 45.18 | 5.23 | 5.43 | 184 | 24 | 9.00 | T(N+)<No | 0.210 | 0.001 |  |
| 2506 | IPI00021298 | KRT20 | Keratin. type I cytoskeletal 20 | 48.51 | 48.6 | 5.52 | 5.6 | 443 | 35 | 14.00 | T(N+)<No | 0.319 | 0.012 | [4] |
| 2509^†^ | IPI00013847 | UQCRC1 | Cytochrome b-c1 complex subunit 1. mitochondrial | 53.30 | 47.09 | 5.94 | 5.73 | 512 | 31 | 18.00 | T(N-)<No | 0.567 | 0.010 | [10] |
|  |  |  |  |  |  |  |  |  |  |  | T(N+)<No | 0.518 | 0.002 |  |
| 2603 | IPI00398625 | HRNR | Hornerin | 28.31 | 52.97 | 10.05 | 5.52 | 217 | 3 | 7.00 | T(N+)<No | 0.509 | 0.030 |  |
|  | IPI00554711 | JUP | Junction plakoglobin | 82.43 |  | 5.75 |  | 141 | 5 | 2.00 |  |  |  |  |
| 2607 | IPI00554648 | KRT8 | Keratin. type II cytoskeletal 8 | 53.67 | 52.27 | 5.52 | 5.65 | 1263 | 53 | 33.00 | T(N+)<No | 0.587 | 0.047 | [3,4,10,11] |
|  |  |  |  |  |  |  |  |  |  |  | T(N+)<T(N-) | 0.382 | 0.007 |  |
| 2803^†^ | IPI00604664 | NDUFS1 | NADH-ubiquinone oxidoreductase 75 kDa subunit | 81.97 | 80.51 | 6.24 | 5.49 | 472 | 39 | 25.00 | T(N-)<No | 0.304 | 0.019 |  |
|  |  |  |  |  |  |  |  |  |  |  | T(N+)<No | 0.466 | 0.050 |  |
| 3103^†^ | IPI00024919 | PRDX3 | Thioredoxin-dependent peroxide reductase. Mitocondrial | 28.02 | 21.07 | 7.67 | 6.33 | 156 | 12 | 3.00 | T(N+)<No | 0.371 | 0.041 | [10] |
|  | IPI00002149 | SAR1B | GTP-binding protein SAR1b | 22.51 |  | 5.76 |  | 156 | 42 | 6.00 |  |  |  |  |
|  | IPI00219757 | GSTP1 | Glutathione S-transferase P | 23.57 |  | 5.43 |  | 149 | 28 | 5.00 |  |  |  | [5,8,11] |
| 3308 | IPI00220740 | NPM1 | Isoform 2 of Nucleophosmin | 29.62 | 33.56 | 4.47 | 6.64 | 108 | 24 | 5.00 | T(N-)<No | * | 0.047 |  |
|  | IPI00218236 | PPP1CB | Serine/threonine-protein phosphatase PP1-beta catalytic subunit | 37.96 |  | 5.84 |  | 98 | 20 | 6.00 |  |  |  |  |
|  | IPI00027423 | PPP1CA | serine/threonine-protein phosphatase PP1-alpha catalytic subunit isoform 3 | 39.41 |  | 6.2 |  | 90 | 19 | 6.00 |  |  |  |  |
|  | IPI00939575 | NAGK | N-acetyl-D-glucosamine kinase | 42.41 |  | 5.81 |  | 44 | 11 | 3.00 |  |  |  |  |
| 3309 | IPI00744692 | TALDO1 | Transaldolase | 37.69 | 32.66 | 6.36 | 6.69 | 124 | 13 | 5.00 | T(N-)>No | 2.325 | 0.009 |  |
|  |  |  |  |  |  |  |  |  |  |  | T(N+)<T(N-) | 0.408 | 0.012 |  |
| 3311 | IPI00006443 | CRYL1 | Isoform 1 of Lambda-crystallin homolog | 35.91 | 29.75 | 5.81 | 6.7 | 389 | 26 | 9.00 | T(N+)<No | 0.197 | 0.005 |  |
|  | IPI00219217 | LDHB | L-lactate dehydrogenase B chain | 36.90 |  | 5.71 |  | 141 | 15 | 5.00 |  |  |  | [10] |
|  | IPI00301109 | PPA2 | Isoform 1 of Inorganic pyrophosphatase 2. mitochondrial | 38.41 |  | 7.07 |  | 93 | 12 | 3.00 |  |  |  |  |
| 3312^†^ | IPI00455154 | GRIN3A | Glutamate [NMDA] receptor subunit 3A | 12.54 | 27.83 | 7.4 | 5.86 | 30 | 0 | 1.00 | T(N+)<No | 0.046 | 0.017 |  |
| 3314^†^ | IPI00398625 | HRNR | Hornerin | 28.31 | 31.79 | 10.05 | 5.92 | 101 | 2 | 4.00 | T(N-)<No | 0.070 | 0.001 |  |
| 3409 | IPI00030702 | IDH3A | Isoform 1 of Isocitrate dehydrogenase [NAD] subunit alpha. mitochondrial | 40.02 | 36.44 | 6.47 | 6 | 156 | 27 | 9.00 | T(N+)<No | 0.446 | 0.030 | [6] |
|  | IPI00019502 | MYH9 | Isoform 1 of Myosin-9 | 22.76 |  | 5.5 |  | 105 | 1 | 2.00 |  |  |  | [1] |
|  | IPI00022434 | ALB | Putative uncharacterized protein ALB | 73.88 |  | 6.33 |  | 42 | 6 | 3.00 |  |  |  |  |
| 3502 | IPI00028091 | ACTR3 | Actin-related protein 3 | 47.80 | 48.2 | 5.61 | 5.82 | 96 | 9 | 4.00 | T(N+)<No | 0.169 | 0.010 |  |
| 3507 | IPI00021435 | PSMC2 | 26S protease regulatory subunit 7 | 49.00 | 47.24 | 5.71 | 6.56 | 493 | 51 | 27.00 | T(N+)>T(N-) | 2.256 | 0.047 |  |
| 3510^†^ | IPI00337494 | SLC25A24 | Isoform 1 of Calcium-binding mitochondrial carrier protein SCaMC-1 | 53.55 | 43.53 | 6 | 6.66 | 254 | 36 | 17.00 | T(N+)<No | 0.432 | 0.032 |  |
| 3608 | IPI00021808 | HARS | Histidyl-tRNA synthetase. cytoplasmic | 57.94 | 52.25 | 5.72 | 6.02 | 206 | 18 | 10.00 | T(N+)>T(N-) | 10.357 | 0.042 |  |
| 4110^†^ | IPI00398727 | LYPLA1 | Isoform 2 of Acyl-protein thioesterase 1 | 23.20 | 20.96 | 6.05 | 6.88 | 79 | 14 | 3.00 | T(N+)<No | 0.318 | 0.013 |  |
| 4201 | IPI00024993 | ECHS1 | Enoyl-CoA hydratase. mitochondrial | 31.82 | 22.49 | 8.34 | 6.72 | 454 | 38 | 12.00 | T(N+)<No | 0.651 | 0.031 | [12] |
| 4205^†^ | IPI00154742 | IGLC2 | IGL protein | 25.12 | 22.62 | 5.93 | 6.78 | 106 | 18 | 6.00 | T(N-)<No | 0.472 | 0.031 |  |
| 4211 | IPI00006092 | PMM2 | Phosphomannomutase 2 | 28.41 | 22.68 | 6.35 | 6.87 | 133 | 27 | 7.00 | T(N+)<No | 0.089 | 0.020 |  |
|  | IPI00154742 | IGLC2 | IGL protein | 25.12 |  | 5.93 |  | 87 | 22 | 4.00 |  |  |  |  |
|  | IPI00643746 | HMGB1 | High-mobility group box 1 | 11.46 |  | 9.91 |  | 34 | 28 | 5.00 |  |  |  |  |
| 4302 | IPI00165360 | MPST | 3-mercaptopyruvate sulfurtransferase | 33.44 | 28.14 | 6.13 | 6.87 | 147 | 38 | 11.00 | T(N+)<T(N-) | 0.343 | 0.032 |  |
|  | IPI00334907 | PITPNB | Isoform 1 of Phosphatidylinositol transfer protein beta isoform | 31.81 |  | 6.41 |  | 63 | 19 | 6.00 |  |  |  |  |
| 4306^†^ | IPI00186008 | STARD10 | PCTP-like protein | 33.43 | 30.92 | 6.67 | 6.79 | 80 | 12 | 3.00 | T(N-)<No | 0.254 | 0.002 |  |
|  | IPI00015809 | OSGEP | Probable O-sialoglycoprotein endopeptidase | 36.92 |  | 5.94 |  | 64 | 8 | 2.00 | T(N+)<No | 0.095 | 0.000 |  |
|  | IPI00219217 | LDHB | L-lactate dehydrogenase B chain | 36.90 |  | 5.71 |  | 64 | 17 | 5.00 |  |  |  | [10] |
| 4401 | IPI00027341 | CAPG | Macrophage-capping protein | 38.78 | 38.97 | 5.88 | 6.72 | 288 | 29 | 11.00 | T(N-)<No | 0.112 | 0.011 |  |
| 4404 | IPI00293102 | PPP2R4 | Isoform 2 of Serine/threonine-protein phosphatase 2A activator | 41.10 | 36.78 | 5.63 | 6.74 | 157 | 33 | 10.00 | T(N-)<No | * | 0.005 |  |
|  |  |  |  |  |  |  |  |  |  |  | T(N+)>T(N-) | * | 0.004 |  |
| 4406 | IPI00008454 | DNAJB11 | DnaJ homolog subfamily B member 11 | 40.77 | 39.9 | 5.81 | 6.77 | 236 | 30 | 12.00 | T(N-)<No | * | 0.008 |  |
|  | IPI00027444 | SERPINB1 | Leukocyte elastase inhibitor | 42.83 |  | 5.9 |  | 172 | 36 | 11.00 |  |  |  |  |
| 4407^†^ | IPI00294158 | BLVRA | Biliverdin reductase A | 33.69 | 34.66 | 6.06 | 6.81 | 167 | 16 | 4.00 | T(N-)>No | 2.020 | 0.035 |  |
| 4409^†^ | IPI00014177 | SEPT2 | Isoform 1 of Septin-2 | 41.69 | 41.29 | 6.15 | 6.84 | 112 | 8 | 4.00 | T(N+)>No | 2.247 | 0.023 |  |
| 4412 | IPI00027444 | SERPINB1 | Leukocyte elastase inhibitor | 42.83 | 38.35 | 5.9 | 6.78 | 660 | 40 | 14.00 | T(N-)>No | 1.907 | 0.011 |  |
|  | IPI00027341 | CAPG | Macrophage-capping protein | 38.78 |  | 5.88 |  | 284 | 14 | 5.00 | T(N+)<T(N-) | 0.535 | 0.046 |  |
|  | IPI00008454 | DNAJB11 | DnaJ homolog subfamily B member 11 | 40.77 |  | 5.81 |  | 106 | 6 | 3.00 |  |  |  |  |
|  | IPI00019383 | GALK1 | cDNA FLJ56840. highly similar to Galactokinase | 45.96 |  | 6.24 |  | 105 | 6 | 3.00 |  |  |  |  |
|  | IPI00383563 | C19orf66 | Isoform 4 of UPF0515 protein C19orf66 | 29.56 |  | 6.47 |  | 52 | 15 | 3.00 |  |  |  |  |
| 4414^†^ | IPI00100933 | PTER | Isoform 1 of Phosphotriesterase-related protein | 39.51 | 36.57 | 6.07 | 6.83 | 155 | 23 | 7.00 | T(N-)<No | 0.226 | 0.004 |  |
|  |  |  |  |  |  |  |  |  |  |  | T(N+)<No | 0.198 | 0.001 |  |
| 4503^†^ | IPI00156689 | VAT1 | Synaptic vesicle membrane protein VAT-1 homolog | 42.12 | 45.36 | 5.88 | 6.74 | 313 | 31 | 9.00 | T(N-)>No | 2.601 | 0.024 |  |
|  |  |  |  |  |  |  |  |  |  |  | T(N+)>No | 2.291 | 0.010 |  |
| 4506 | IPI00002460 | ANXA7 | Isoform 1 of Annexin A7 | 52.99 | 46.12 | 5.52 | 6.78 | 168 | 26 | 12.00 | T(N-)<No | * | 0.026 |  |
|  | IPI00215952 | ENTPD5 | Ectonucleoside triphosphate diphosphohydrolase 5 | 47.89 |  | 5.92 |  | 37 | 6 | 2.00 |  |  |  |  |
| 4606^†^ | IPI00218914 | ALDH1A1 | Retinal dehydrogenase 1 | 55.45 | 53.81 | 6.3 | 6.8 | 260 | 25 | 13.00 | T(N-)<No | * | 0.011 | [1] |
|  | IPI00180675 | TUBA1A | Tubulin alpha-1A chain | 50.79 |  | 4.94 |  | 85 | 19 | 6.00 | T(N+)<No | 0.079 | 0.023 | [5] |
|  | IPI00297779 | CCT2 | T-complex protein 1 subunit beta | 57.79 |  | 6.01 |  | 81 | 11 | 6.00 |  |  |  |  |
| 4610^†^ | IPI00218914 | ALDH1A1 | Retinal dehydrogenase 1 | 55.45 | 54.11 | 6.30 | 6.86 | 334 | 22 | 8.00 | T(N+)<No | 0.370 | 0.017 | [1] |
| 4611 | IPI00009032 | SSB | Lupus La protein | 46.98 | 49.56 | 6.68 | 6.78 | 138 | 14 | 5.00 | T(N+)>T(N-) | * | 0.050 |  |
|  | IPI00022434 | ALB | Putative uncharacterized protein ALB | 73.88 |  | 6.33 |  | 91 | 7 | 5.00 |  |  |  |  |
| 4612^†^ | IPI00003944 | DBT | Lipoamide acyltransferase component of branched-chain alpha-keto acid dehydrogenase complex. Mitochondrial | 53.85 | 50.44 | 8.71 | 6.88 | 257 | 35 | 17.00 | T(N-)<No | * | 0.013 | [10] |
|  |  |  |  |  |  |  |  |  |  |  | T(N+)<No | 0.082 | 0.026 |  |
| 4701 | IPI00019812 | PPP5C | Serine/threonine-protein phosphatase 5 | 57.41 |  | 5.88 |  | 69 | 10 | 3.00 | T(N+)>T(N-) | * | 0.039 |  |
| 4716 | IPI00029111 | DPYSL3 | Collapsin response mediator protein 4 long variant | 74.32 | 58.23 | 5.94 | 6.87 | 504 | 33 | 17.00 | T(N+)>No | 4.325 | 0.039 |  |
| 4717^†^ | IPI00257508 | DPYSL2 | Dihydropyrimidinase-related protein 2 | 62.71 | 60.56 | 5.95 | 6.83 | 320 | 21 | 12.00 | T(N+)>No | 1.928 | 0.003 |  |
| 4804 | IPI00783097 | GARS | Glycyl-tRNA synthetase | 83.83 | 76.8 | 6.61 | 6.75 | 250 | 22 | 16.00 | T(N-)<No | * | 0.024 |  |
| 4806 | IPI00843975 | EZR | Ezrin | 69.48 | 85.36 | 5.94 | 6.78 | 254 | 32 | 21.00 | T(N-)<No | 0.125 | 0.003 | [5] |
|  | IPI00218694 | CALD1 | Isoform 2 of Caldesmon | 65.73 |  | 6.37 |  | 191 | 26 | 16.00 |  |  |  |  |
|  | IPI00553177 | SERPINA1 | Isoform 1 of Alpha-1-antitrypsin | 46.88 |  | 5.37 |  | 52 | 12 | 4.00 |  |  |  | [4,13] |
| 4808^†^ | IPI00843975 | EZR | Ezrin | 69.48 | 84.02 | 5.94 | 6.81 | 821 | 34 | 27.00 | T(N-)<No | 0.543 | 0.012 | [5] |
| 4904^†^ | IPI00921523 | CFB | Complement factor B | 86.85 | 119.81 | 6.67 | 6.83 | 144 | 15 | 12.00 | T(N+)>No | 3.576 | 0.030 |  |
| 5102^†^ | IPI00026964 | UQCRFS1 | Cytochrome b-c1 complex subunit Rieske. mitochondrial | 29.93 | 21.55 | 8.55 | 6.95 | 427 | 27 | 13.00 | T(N-)<No | 0.562 | 0.012 |  |
|  |  |  |  |  |  |  |  |  |  |  | T(N+)<No | 0.318 | 0.000 |  |
|  |  |  |  |  |  |  |  |  |  |  | T(N+)<T(N-) | 0.517 | 0.032 |  |
| 5303^†^ | IPI00553131 | GALE | UDP-glucose 4-epimerase | 38.66 | 32.61 | 6.26 | 6.9 | 264 | 18 | 6.00 | T(N+)<No | 0.123 | 0.001 | [10] |
| 5305^†^ | IPI00218918 | ANXA1 | Annexin A1 | 38.92 | 32.75 | 6.57 | 6.93 | 642 | 38 | 20.00 | T(N+)>No | 1.668 | 0.031 | [6,10] |
| 5310^†^ | IPI00219617 | PRPS2 | Isoform 1 of Ribose-phosphate pyrophosphokinase 2 | 35.15 | 28.5 | 6.15 | 6.92 | 278 | 37 | 12.00 | T(N+)<No | 0.163 | 0.004 |  |
| 5401 | IPI00909944 | ACADS | cDNA FLJ51323. highly similar to Short-chain specific acyl-CoA dehydrogenase. Mitochondrial | 44.50 | 38.39 | 8.65 | 6.9 | 299 | 43 | 13.00 | T(N+)<No | 0.417 | 0.031 |  |
| 5409^†^ | IPI00147874 | NANS | Sialic acid synthase | 40.74 | 37.82 | 6.29 | 7.1 | 423 | 35 | 13.00 | T(N+)<No | 0.481 | 0.018 | [10] |
| 5502^†^ | IPI00025239 | NDUFS2 | NADH dehydrogenase [ubiquinone] iron-sulfur protein 2. mitochondrial | 52.91 | 44.87 | 7.21 | 6.92 | 199 | 14 | 8.00 | T(N-)<No | 0.306 | 0.037 |  |
|  |  |  |  |  |  |  |  |  |  |  | T(N+)<No | 0.214 | 0.005 |  |
| 5505^†^ | IPI00010130 | GLUL | Glutamine synthetase | 42.67 | 42.83 | 6.43 | 6.95 | 386 | 26 | 12.00 | T(N-)<No | 0.073 | 0.000 |  |
|  |  |  |  |  |  |  |  |  |  |  | T(N+)<No | 0.116 | 0.000 |  |
| 5506^†^ | IPI00465248 | ENO1 | Isoform alpha-enolase of Alpha-enolase | 47.48 | 48.17 | 7.01 | 7.05 | 708 | 30 | 17.00 | T(N+)>No | 2.634 | 0.006 | [1,4,5,10,11] |
| 5602^†^ | IPI00009893 | LIPF | cDNA FLJ55598. highly similar to Gastric triacylglycerol lipase | 46.50 | 50.6 | 8.21 | 6.9 | 161 | 17 | 7.00 | T(N-)<No | 0.046 | 0.014 | [10] |
|  |  |  |  |  |  |  |  |  |  |  | T(N+)<No | 0.015 | 0.011 |  |
| 5604^†^ | IPI00218914 | ALDH1A1 | Retinal dehydrogenase 1 | 55.45 | 53.6 | 6.30 | 6.93 | 918 | 40 | 21.00 | T(N-)<No | 0.413 | 0.049 | [1] |
|  |  |  |  |  |  |  |  |  |  |  | T(N+)<No | 0.376 | 0.011 |  |
| 5606^†^ | IPI00009893 | LIPF | cDNA FLJ55598. highly similar to Gastric triacylglycerol lipase | 46.50 | 50.14 | 8.21 | 7 | 233 | 17 | 10.00 | T(N-)<No | * | 0.001 | [10] |
|  |  |  |  |  |  |  |  |  |  |  | T(N+)<No | 0.005 | 0.001 |  |
| 5703 | IPI00010133 | CORO1A | Coronin-1A | 51.68 | 56.77 | 6.25 | 6.9 | 348 | 18 | 10.00 | T(N+)>T(N-) | 4.351 | 0.037 | [10] |
| 5707^†^ | IPI00298497 | FGB | Fibrinogen beta chain | 56.58 | 56.7 | 8.54 | 6.95 | 220 | 13 | 7.00 | T(N+)>No | 2.112 | 0.023 | [9] |
| 5708^†^ | IPI00013894 | STIP1 | Stress-induced-phosphoprotein 1 | 63.23 | 61.52 | 6.40 | 6.96 | 377 | 21 | 13.00 | T(N+)>No | 2.394 | 0.020 |  |
| 5710 | IPI00291510 | IMPDH2 | Inosine-5'-monophosphate dehydrogenase 2 | 56.23 | 55.75 | 6.44 | 7.04 | 238 | 35 | 17.00 | T(N-)<No | * | 0.036 |  |
|  | IPI00216008 | G6PD | Isoform Long of Glucose-6-phosphate 1-dehydrogenase | 64.30 |  | 6.43 |  | 52 | 5 | 3.00 |  |  |  |  |
| 5801^†^ | IPI00219365 | MSN | Moesin | 67.89 | 74.51 | 6.08 | 6.89 | 570 | 41 | 32.00 | T(N+)>No | 2.029 | 0.032 |  |
|  | IPI00021405 | LMNA | Isoform A of Lamin-A/C | 74.38 |  | 6.57 |  | 206 | 20 | 13.00 |  |  |  |  |
|  | IPI00384282 | EZR | Cytovillin 2 (Fragment) | 16.29 |  | 9.32 |  | 99 | 33 | 10.00 |  |  |  | [5] |
| 5803 | IPI00745872 | ALB | Isoform 1 of Serum albumin | 71.32 | 64.17 | 5.92 | 6.9 | 515 | 21 | 14.00 | T(N-)<No | * | 0.010 | [3,5,12,13] |
| 5902^†^ | IPI00186290 | EEF2 | Elongation factor 2 | 96.25 | 114.33 | 6.41 | 6.99 | 372 | 36 | 37.00 | T(N+)<No | 0.366 | 0.023 | [5,10] |
| 6104^†^ | IPI00329266 | GKN2 | Gastrokine-2 | 20.76 | 18.37 | 7.03 | 7.58 | 201 | 26 | 7.00 | T(N-)<No | * | 0.006 |  |
|  |  |  |  |  |  |  |  |  |  |  | T(N+)<No | 0.052 | 0.003 |  |
| 6304^†^ | IPI00293721 | AKR7A3 | Aflatoxin B1 aldehyde reductase member 3 | 37.58 | 32.61 | 6.67 | 7.32 | 351 | 33 | 11.00 | T(N-)<No | 0.156 | 0.013 | [10] |
|  |  |  |  |  |  |  |  |  |  |  | T(N+)<No | 0.137 | 0.003 |  |
| 6305 | IPI00010810 | ETFA | Electron transfer flavoprotein subunit alpha. mitochondrial | 35.40 | 25.93 | 8.62 | 7.42 | 152 | 15 | 6.00 | T(N-)<No | 0.549 | 0.034 |  |
| 6306^†^ | IPI00418169 | ANXA2 | Isoform 2 of Annexin A2 | 40.67 | 31.93 | 8.53 | 7.45 | 428 | 36 | 12.00 | T(N+)>No | 4.042 | 0.049 | [5,11] |
| 6308^†^ | IPI00015262 | CNN2 | Calponin-2 | 34.07 | 30.31 | 6.95 | 7.31 | 91 | 23 | 6.00 | T(N+)>No | 9.829 | 0.028 |  |
|  | IPI00916111 | MDH1 | Malate dehydrogenase | 38.92 |  | 8.11 |  | 81 | 15 | 5.00 |  |  |  | [5,10] |
| 6403^†^ | IPI00219029 | GOT1 | Aspartate aminotransferase. Cytoplasmic | 46.45 | 40.63 | 6.52 | 7.44 | 379 | 26 | 9.00 | T(N-)<No | 0.217 | 0.002 | [10] |
|  |  |  |  |  |  |  |  |  |  |  | T(N+)<No | 0.221 | 0.001 |  |
| 6502^†^ | IPI00922697 | PDHA1 | Pyruvate dehydrogenase E1 component subunit alpha. somatic form. mitochondrial | 43.95 | 43.32 | 8.35 | 7.23 | 315 | 22 | 9.00 | T(N-)<No | 0.290 | 0.002 |  |
|  |  |  |  |  |  |  |  |  |  |  | T(N+)<No | 0.277 | 0.000 |  |
| 6503 | IPI00008934 | HMGCS2 | Hydroxymethylglutaryl-CoA synthase. mitochondrial | 57.11 | 48 | 8.40 | 7.27 | 379 | 18 | 12.00 | T(N-)>No | 5.680 | 0.010 |  |
|  |  |  |  |  |  |  |  |  |  |  | T(N+)<T(N-) | 0.205 | 0.024 |  |
| 6505^†^ | IPI00465248 | ENO1 | Isoform alpha-enolase of Alpha-enolase | 47.48 | 47.95 | 7.01 | 7.41 | 1449 | 53 | 29.00 | T(N-)>No | 1.578 | 0.017 | [1,4,5,10,11] |
|  |  |  |  |  |  |  |  |  |  |  | T(N+)>No | 1.854 | 0.048 |  |
| 6602^†^ | IPI00009893 | LIPF | cDNA FLJ55598. highly similar to Gastric triacylglycerol lipase | 46.50 | 50.01 | 8.21 | 7.26 | 249 | 17 | 10.00 | T(N-)<No | * | 0.001 | [10] |
|  |  |  |  |  |  |  |  |  |  |  | T(N+)<No | ** | 0.001 |  |
| 6609^†^ | IPI00009893 | LIPF | cDNA FLJ55598. highly similar to Gastric triacylglycerol lipase | 46.50 | 50 | 8.21 | 7.56 | 268 | 17 | 12.00 | T(N-)<No | * | 0.002 | [10] |
|  |  |  |  |  |  |  |  |  |  |  | T(N+)<No | ** | 0.002 |  |
| 6703^†^ | IPI00298497 | FGB | Fibrinogen beta chain | 56.58 | 56.66 | 8.54 | 7.2 | 382 | 37 | 17.00 | T(N-)>No | 1.785 | 0.043 | [9] |
| 6706^†^ | IPI00015911 | DLD | Dihydrolipoyl dehydrogenase. mitochondrial | 54.71 | 56.12 |  | 7.32 |  |  | 10.00 | T(N-)<No | 0.528 | 0.017 | [2] |
| 7102^†^ | IPI00024915 | PRDX5 | Isoform Mitochondrial of Peroxiredoxin-5. mitochondrial | 22.30 | 17.08 | 8.93 | 7.65 | 120 | 17 | 3.00 | T(N-)<No | 0.106 | 0.001 | [3,12] |
|  |  |  |  |  |  |  |  |  |  |  | T(N+)<No | 0.200 | 0.000 |  |
| 7103 | IPI00022314 | SOD2 | Superoxide dismutase [Mn]. mitochondrial | 24.88 | 20.39 | 8.35 | 7.66 | 293 | 27 | 11.00 | T(N+)>No | 1.625 | 0.043 |  |
| 7104^†^ | IPI00783862 | BLVRB | Flavin reductase | 22.22 | 21.1 | 7.13 | 7.92 | 129 | 35 | 5 | T(N-)<No | 0.139 | 0.008 | [12] |
|  |  |  |  |  |  |  |  |  |  |  | T(N+)<No | 0.203 | 0.016 |  |
| 7107^†^ | IPI00783862 | BLVRB | Flavin reductase | 22.22 | 21.11 | 7.13 | 8.21 | 327 | 38 | 8.00 | T(N+)<No | 0.449 | 0.021 | [12] |
| 7202^†^ | IPI00218414 | CA2 | Carbonic anhydrase 2 | 29.29 | 24.13 | 6.87 | 7.66 | 386 | 43 | 15.00 | T(N-)<No | * | 0.012 | [1,4,5,11] |
|  |  |  |  |  |  |  |  |  |  |  | T(N+)<No | 0.035 | 0.016 |  |
| 7203^†^ | IPI00218414 | CA2 | Carbonic anhydrase 2 | 29.29 | 22.88 | 6.87 | 7.67 | 427 | 47 | 16.00 | T(N-)<No | 0.268 | 0.000 | [1,4,5,11] |
|  |  |  |  |  |  |  |  |  |  |  | T(N+)<No | 0.374 | 0.000 |  |
| 7303^†^ | IPI00105407 | AKR1B10 | Aldo-keto reductase family 1 member B10 | 36.23 | 31.5 | 7.12 | 7.85 | 598 | 37 | 17.00 | T(N-)<No | 0.370 | 0.014 | [6] |
|  |  |  |  |  |  |  |  |  |  |  | T(N+)<No | 0.284 | 0.003 |  |
| 7401^†^ | IPI00030207 | GMDS | GDP-mannose 4.6 dehydratase | 42.27 | 40.35 | 6.87 | 7.62 | 152 | 16 | 6.00 | T(N+)<No | 0.332 | 0.002 |  |
| 7402^†^ | IPI00029561 | NDUFA10 | NADH dehydrogenase [ubiquinone] 1 alpha subcomplex subunit 10. mitochondrial | 41.07 | 37.4 | 8.67 | 7.67 | 89 | 13 | 4.00 | T(N-)<No | 0.137 | 0.004 | [2] |
|  | IPI00398625 | HRNR | Hornerin | 28.31 |  | 10.05 |  | 35 | 2 | 4.00 | T(N+)<No | 0.184 | 0.005 |  |
| 7407^†^ | IPI00030363 | ACAT1 | Acetyl-CoA acetyltransferase. mitochondrial | 45.46 | 40.95 | 8.89 | 8.08 | 235 | 41 | 15.00 | T(N+)<No | 0.185 | 0.005 |  |
|  | IPI00028888 | HNRNPD | Isoform 1 of Heterogeneous nuclear ribonucleoprotein D0 | 38.58 |  | 7.62 |  | 60 | 6 | 2.00 |  |  |  |  |
| 7712^†^ | IPI00028031 | ACADVL | cDNA FLJ56425. highly similar to Very-long-chain specific acyl-CoAdehydrogenase. mitochondrial | 75.62 | 61.91 | 9.16 | 8.24 | 602 | 33 | 25.00 | T(N-)<No | 0.251 | 0.024 |  |
| 7805 | IPI00000690 | AIFM1 | Isoform 1 of Apoptosis-inducing factor 1. mitochondrial | 67.14 | 63.5 | 9.04 | 7.85 | 246 | 25 | 15.00 | T(N-)<No | * | 0.028 | [10] |
| 7809^†^ | IPI00000690 | AIFM1 | Isoform 1 of Apoptosis-inducing factor 1. mitochondrial | 67.14 | 63.19 | 9.04 | 8.13 | 497 | 25 | 14.00 | T(N-)<No | 0.208 | 0.007 | [10] |
|  |  |  |  |  |  |  |  |  |  |  | T(N+)<No | 0.092 | 0.001 |  |
| 7901^†^ | IPI00017855 | ACO2 | Aconitate hydratase. mitochondrial | 86.11 | 96.15 | 7.36 | 7.65 | 165 | 6 | 4.00 | T(N+)<No | 0.065 | 0.024 | [5,10] |
| 7904^†^ | IPI00017855 | ACO2 | Aconitate hydratase. mitochondrial | 86.11 | 95.1 | 7.36 | 7.82 | 464 | 19 | 15.00 | T(N-)<No | * | 0.010 | [5,10] |
|  |  |  |  |  |  |  |  |  |  |  | T(N+)<No | 0.028 | 0.012 |  |
| 8109^†^ | IPI00219034 | NDUFA8 | NADH dehydrogenase [ubiquinone] 1 alpha subcomplex subunit 8 | 20.55 | 18.79 | 7.57 | 8.53 | 112 | 31 | 6.00 | T(N-)<No | 0.071 | 0.003 |  |
|  |  |  |  |  |  |  |  |  |  |  | T(N+)<No | 0.107 | 0.005 |  |
| 8206^†^ | IPI00004902 | ETFB | Isoform 1 of Electron transfer flavoprotein subunit beta | 28.05 | 23.35 | 8.24 | 8.96 | 422 | 44 | 12.00 | T(N+)<No | 0.243 | 0.016 | [11] |
| 8208^†^ | IPI00215901 | AK2 | Isoform 1 of Adenylate kinase 2. mitochondrial | 26.69 | 23.63 | 7.67 | 8.52 | 161 | 56 | 11.00 | T(N-)<No | 0.281 | 0.033 |  |
|  | IPI00171199 | PSMA3 | Isoform 2 of Proteasome subunit alpha type-3 | 27.86 |  | 5.19 |  | 76 | 14 | 3.00 | T(N+)<No | 0.094 | 0.000 |  |
|  | IPI00465431 | LGALS3 | Galectin-3 | 26.19 |  | 8.57 |  | 38 | 13 | 3.00 |  |  |  | [5] |
| 8302 | IPI00219018 | GAPDH | Glyceraldehyde-3-phosphate dehydrogenase | 36.20 | 33.94 | 8.57 | 8.45 | 174 | 22 | 7.00 | T(N+)<No | 0.092 | 0.042 | [5] |
|  | IPI00029733 | AKR1C1 | Aldo-keto reductase family 1 member C1 | 37.22 | 33.94 | 8.02 | 8.45 | 166 | 23 | 7.00 |  |  |  |  |
| 8309^†^ | IPI00216308 | VDAC1 | Voltage-dependent anion-selective channel protein 1 | 30.87 | 27.21 | 8.62 | 9.09 | 261 | 21 | 7.00 | T(N-)<No | 0.313 | 0.011 | [1,11] |
|  |  |  |  |  |  |  |  |  |  |  | T(N+)<No | 0.264 | 0.002 |  |
| 8310^†^ | IPI00294398 | HADH | Isoform 1 of Hydroxyacyl-coenzyme A dehydrogenase. Mitochondrial | 34.33 | 27.41 | 8.88 | 9.18 | 370 | 26 | 11.00 | T(N-)<No | 0.493 | 0.024 | [3] |
|  |  |  |  |  |  |  |  |  |  |  | T(N+)<No | 0.487 | 0.008 |  |
| 8402^†^ | IPI00030363 | ACAT1 | Acetyl-CoA acetyltransferase. mitochondrial | 45.46 | 40.95 | 8.98 | 8.57 | 443 | 31 | 15.00 | T(N-)<No | 0.276 | 0.001 |  |
|  |  |  |  |  |  |  |  |  |  |  | T(N+)<No | 0.199 | 0.000 |  |
| 8404^†^ | IPI00021439 | ACTB | Actin. cytoplasmic 1 | 42.05 | 38.22 | 5.29 | 8.85 | 51 | 14 | 3.00 | T(N-)<No | * | 0.001 | [5,10,11] |
|  | IPI00396258 | BCAT2 | Isoform A of Branched-chain-amino-acid aminotransferase. mitochondrial | 44.66 |  | 8.88 |  | 49 | 9 | 4.00 | T(N+)<No | 0.137 | 0.010 |  |
| 8407^†^ | IPI00396258 | BCAT2 | Isoform A of Branched-chain-amino-acid aminotransferase. mitochondrial | 44.66 | 37.91 | 8.88 | 8.42 | 68 | 10 | 5.00 | T(N-)<No | * | 0.011 |  |
|  |  |  |  |  |  |  |  |  |  |  | T(N+)<No | ** | 0.011 |  |
| 8501^†^ | IPI00305383 | UQCRC2 | Cytochrome b-c1 complex subunit 2. mitochondrial | 48.58 | 44.24 | 8.74 | 8.34 | 220 | 17 | 13.00 | T(N-)<No | 0.317 | 0.016 |  |
|  | IPI00011107 | IDH2 | Isocitrate dehydrogenase [NADP]. mitochondrial | 51.33 |  | 8.88 |  | 351 | 33 | 9.00 | T(N+)<No | 0.174 | 0.001 | [6] |
| 8505^†^ | IPI00011107 | IDH2 | Isocitrate dehydrogenase [NADP]. mitochondrial | 51.33 | 44.66 | 8.88 | 8.66 | 584 | 27 | 22.00 | T(N-)<No | 0.240 | 0.014 | [6] |
|  |  |  |  |  |  |  |  |  |  |  | T(N+)<No | 0.354 | 0.020 |  |
| 8506^†^ | IPI00011107 | IDH2 | Isocitrate dehydrogenase [NADP]. mitochondrial | 51.33 | 44.75 | 8.88 | 8.91 | 814 | 31 | 22.00 | T(N+)<No | 0.304 | 0.009 | [6] |
| 8513 | IPI00383539 | CS | Citrate synthase | 47.19 | 44.15 | 6.74 | 8.14 | 135 | 19 | 5.00 | T(N-)<No | * | 0.012 | [5] |
| 8606^†^ | IPI00024990 | ALDH6A1 | Methylmalonate-semialdehyde dehydrogenase [acylating]. mitochondrial | 58.26 | 54.84 | 8.72 | 8.48 | 196 | 8 | 5.00 | T(N-)<No | * | 0.016 |  |
|  | IPI00440493 | ATP5A1 | ATP synthase subunit alpha. mitochondrial | 59.83 |  | 9.16 |  | 151 | 9 | 4.00 | T(N+)<No | ** | 0.016 |  |
|  | IPI00384938 | IGHV4-31 | Putative uncharacterized protein DKFZp686N02209 | 53.50 |  | 8.74 |  | 61 | 6 | 2.00 |  |  |  |  |
| 8614^†^ | IPI00011107 | IDH2 | Isocitrate dehydrogenase [NADP]. mitochondrial | 50.88 | 46.03 | 8.88 | 8.34 | 297 | 29 | 12.00 | T(N-)<No | * | 0.016 | [6] |
|  |  |  |  |  |  |  |  |  |  |  | T(N+)<No | 0.030 | 0.020 |  |
| 8707 | IPI00908881 | GPI | Glucose-6-phosphate isomerase | 60.40 | 56.61 | 8.61 | 9.16 | 149 | 9 | 6.00 | T(N-)<No | * | 0.001 |  |
|  |  |  |  |  |  |  |  |  |  |  | T(N+)>T(N-) | * | 0.012 |  |
| 9205^†^ | IPI00657682 | GSTA1 | Glutathione S-transferase A1 | 25.67 | 22.06 | 8.91 | 10 | 238 | 27 | 9.00 | T(N+)<No | 0.090 | 0.019 | [5,8,11] |
| 9302^†^ | IPI00396378 | HNRNPA2B1 | Isoform B1 of Heterogeneous nuclear ribonucleoproteins A2/B1 | 37.46 | 32.01 | 8.97 | 9.27 | 287 | 24 | 9.00 | T(N+)<No | 0.356 | 0.017 | [5,6,11] |
|  | IPI00291006 | MDH2 | Malate dehydrogenase. mitochondrial | 35.94 |  | 8.92 |  | 67 | 12 | 3.00 |  |  |  | [5,10] |
| 9305^†^ | IPI00216308 | VDAC1 | Voltage-dependent anion-selective channel protein 1 | 30.87 | 27.13 | 8.62 | 9.4 | 523 | 43 | 12.00 | T(N-)<No | 0.598 | 0.041 | [1,11] |
|  |  |  |  |  |  |  |  |  |  |  | T(N+)<No | 0.609 | 0.022 |  |
| 9310 | IPI00012069 | NQO1 | NAD(P)H dehydrogenase [quinone] 1 | 30.91 | 24.64 | 8.91 | 10 | 59 | 13 | 3.00 | T(N+)<No | 0.036 | 0.014 |  |

^†^Differentially expressed in the paired T-test analysis; *Absent in tumor without lymph node metastasis; **Absent in tumor with lymph node metastasis; ^ǂ^ Previous proteomic studies of gastric tumor that identified the related protein as differentially expressed. In some cases, a previous study identified proteins without description of isoforms and these proteins were also included in the present list, e.g. some studies identified malate dehydrogenase and these were listed in the table as related to MDH1 and MDH2. No: non-neoplastic samples; T(N-): tumors without lymph node metastasis; T(N+): tumors with lymph node metastasis; Theor: Theoretical; Exp: Experimental.

1. Bai Z, Ye Y, Liang B, Xu F, Zhang H, et al. (2011) Proteomics-based identification of a group of apoptosis-related proteins and biomarkers in gastric cancer. Int J Oncol 38: 375-383.

2. Ebert MP, Rocken C (2006) Molecular screening of gastric cancer by proteome analysis. Eur J Gastroenterol Hepatol 18: 847-853.

3. Li N, Zhang J, Liang Y, Shao J, Peng F, et al. (2007) A controversial tumor marker: is SM22 a proper biomarker for gastric cancer cells? J Proteome Res 6: 3304-3312.

4. He QY, Cheung YH, Leung SY, Yuen ST, Chu KM, et al. (2004) Diverse proteomic alterations in gastric adenocarcinoma. Proteomics 4: 3276-3287.

5. Yoshihara T, Kadota Y, Yoshimura Y, Tatano Y, Takeuchi N, et al. (2006) Proteomic alteration in gastic adenocarcinomas from Japanese patients. Mol Cancer 5: 75.

6. Zhang J, Kang B, Tan X, Bai Z, Liang Y, et al. (2007) Comparative analysis of the protein profiles from primary gastric tumors and their adjacent regions: MAWBP could be a new protein candidate involved in gastric cancer. J Proteome Res 6: 4423-4432.

7. Jang JS, Cho HY, Lee YJ, Ha WS, Kim HW (2004) The differential proteome profile of stomach cancer: identification of the biomarker candidates. Oncol Res 14: 491-499.

8. Nishigaki R, Osaki M, Hiratsuka M, Toda T, Murakami K, et al. (2005) Proteomic identification of differentially-expressed genes in human gastric carcinomas. Proteomics 5: 3205-3213.

9. Wang KJ, Wang RT, Zhang JZ (2004) Identification of tumor markers using two-dimensional electrophoresis in gastric carcinoma. World J Gastroenterol 10: 2179-2183.

10. Cai Z, Zhao JS, Li JJ, Peng DN, Wang XY, et al. (2010) A combined proteomics and metabolomics profiling of gastric cardia cancer reveals characteristic dysregulations in glucose metabolism. Mol Cell Proteomics 9: 2617-2628.

11. Cheng Y, Zhang J, Li Y, Wang Y, Gong J (2007) Proteome analysis of human gastric cardia adenocarcinoma by laser capture microdissection. BMC Cancer 7: 191.

12. Wu C, Luo Z, Chen X, Wu C, Yao D, et al. (2009) Two-dimensional differential in-gel electrophoresis for identification of gastric cancer-specific protein markers. Oncol Rep 21: 1429-1437.

13. Ryu JW, Kim HJ, Lee YS, Myong NH, Hwang CH, et al. (2003) The proteomics approach to find biomarkers in gastric cancer. J Korean Med Sci 18: 505-509.
